# Design of a live-attenuated bacterial vaccine using effector network engineering

**DOI:** 10.1038/s41467-026-76138-7

**Published:** 2026-08-07

**Authors:** Sarah Jordan, Priyanka Biswas, Maria L. Alberdi, Jake Samuel, Zuza Kozic, Rita Berkachy, Vishwas Mishra, Nazila Kamaly, Oscar Ces, Jyoti Choudhary, Julia Sanchez-Garrido, Abigail Clements, Gad Frankel

**Affiliations:** 1 Department of Life Sciences, Imperial College London, London, UK; 2 Department of Chemistry, Imperial College London, London, UK; 3 Functional Proteomics Group, Chester Beatty Laboratories, Institute of Cancer Research, London, UK

**Keywords:** Live attenuated vaccines, Bacterial pathogenesis, Live attenuated vaccines

## Abstract

Enteropathogenic *Escherichia coli* (EPEC) and *Citrobacter rodentium* (CR) are extracellular enteric attaching and effacing (A/E) pathogens of humans and mice, respectively. Their virulence relies on intimate bacterial attachment and a network of type III secretion system effectors. Here, through systematic reduction and redesign of the effector network in CR, we develop an attenuated strain, CRV (CR Vaccine), encoding a subset of ten effectors. CRV colonises C57BL/6 mice ~100-fold lower than wild type CR (CR_WT_) without causing overt pathogenesis. Moreover, C3H/HeN mice, which succumb to CR_WT_ infection, survive CRV challenge. Vaccination with CRV confers protection against subsequent CR_WT_ infection in both mouse strains. Serological analysis reveals a repertoire of dominant CR antigens, including the O-antigen, the virulence factors intimin, EspA and Tir and the outer membrane proteins Lpp, OmpA, MetQ and CARC (an AIDA-like autotransporter). We show that CR_WT_ and CRV immunisation elicits comparable B cell and antibody responses, which is contingent on intimate bacterial attachment. A corresponding EPEC strain (*E. coli* Vaccine, ECV) effectively colonises epithelial cells in a gut-on-chip model. These findings establish effector network minimisation as a generalisable strategy for rational bacterial attenuation and live-attenuated vaccine design.

## Abstract (web summary)

Here, the authors generate an attenuated vaccine of Citrobacter rodentium through systematic reduction and redesign of the effector network, which can potentially serve as a path towards the development of live-attenuated vaccines against diarrhoeal disease.

## Introduction

Live attenuated vaccines (LAVs) are a proven strategy for preventing infection. Their replication competency enables efficient antigen presentation and durable protective immunity, often eliminating the need for multiple boosters or adjuvants. Moreover, mucosal delivery of LAVs promotes immune activation at the relevant infection niche^1^. While many viral LAVs are widely used, development of bacterial LAVs has lagged, in part due to the challenge of achieving safety without compromising immunogenicity^2^. However, advances in the understanding of bacterial pathogenesis and progress in molecular genetics now enable rational attenuation strategies beyond classical approaches such as serial passage (e.g., BCG^3^).

Enteropathogenic and enterohaemorrhagic *Escherichia coli* (EPEC and EHEC) are extracellular enteric pathogens that significantly contribute to the global burden of diarrhoeal disease. EPEC is a leading cause of infant mortality due to diarrhoea in the developing world^4^. EHEC is a zoonotic pathogen that infects both humans and ruminants, with outbreaks reported worldwide^5^. Infection with Shiga toxin (Stx)-producing EHEC serotypes, such as O157:H7, can result in haemorrhagic colitis and life-threatening sequelae, including haemolytic uremic syndrome (HUS)^6,7^. Importantly, antibiotic use is contraindicated in EHEC infection as it can induce increased Stx release^8^. Vaccination remains a promising strategy for limiting disease burden and zoonotic transmission of EPEC and EHEC^9^, however only one licensed example exists. This vaccine utilises a siderophore receptor/protein (SRP) technology to limit EHEC shedding in cattle and is only licensed for use within the United States^10^.

EPEC, EHEC and the related murine pathogen *Citrobacter rodentium* (CR) employ a conserved infection strategy characterised by the formation of attaching and effacing (A/E) lesions. These lesions enable intimate bacterial adherence to host intestinal epithelial cells (IEC), effacement of the microvilli brush border and formation of actin-rich pedestal-like structures^11,12^. A/E pathogenesis is orchestrated by a polycistronic pathogenicity island known as the locus of enterocyte effacement (LEE)^13^. The LEE encodes the structural components of a type III secretion system (T3SS), regulatory proteins (e.g., Ler), chaperones (e.g. CesT), the outer membrane adhesin intimin and the translocated effectors EspF, EspG, EspH, EspZ, Map and Tir. A/E lesion formation is initiated by the injection of effectors through the T3SS into host cells, starting with translocated intimin receptor (Tir), which integrates into the plasma membrane of IECs in a hairpin loop topology and binds intimin^14,15^. Other effectors, encoded both on the LEE and within prophage and insertion genomic segments, hijack key signalling processes within host cells. These include actin cytoskeletal rearrangements (e.g., EspH, a RhoGEF inhibitor)^16,17^, tight junction disassembly (e.g., EspF)^18,19^ and inflammatory signalling inhibition (e.g., NleC, a zinc-metalloprotease that cleaves NF-κB p65)^20^. While facilitating colonisation, effector activity drives intestinal pathology. Effector repertoires vary across clinical isolates, for example, EPEC and EHEC strains encode between 21 and 50 effectors, while CR encodes 31^21^. As mice are inherently resistant to EPEC and EHEC infection^22^, CR is the gold-standard model for studying A/E pathogenesis and effector function in vivo^23,24^.

CR infection severity is determined by the host genetic background: while C57BL/6 and BALB/c mice develop self-limiting disease, C3H/HeN and FVB mice are susceptible and succumb to infection^25,26^. In resistant C57BL/6 mice, CR initially colonises the caecal patch before disseminating along the distal colon^27^. Bacterial shedding peaks around 8 days post-infection (dpi), reaching ~ 10^9^ colony-forming units per gram of faeces (CFU/GoF) and triggers colitis marked by intestinal inflammation and colonic crypt hyperplasia (CCH)^28^. Clearance of mucosal-associated CR begins around 12 dpi and is mediated by mucosal IgG and opsonophagocytosis; indeed, μMT mice lacking mature B cells cannot clear the infection^29,30^. Infection resolves by ~ 21 dpi, with the luminal population outcompeted by the gut microbiota, and results in protective immunity^31^. Prior studies have shown that a subset of virulence factors comprise the immunodominant antigens recognised during infection, specifically intimin and the T3SS translocator EspA^31^.

Uncovering individual effector roles is complicated, as many single-gene deletions fail to produce discernible phenotypic changes. Further difficulty arises from synergistic and antagonistic functions, for example, EspM and Map both mimic RhoA GEFs, while NleC and NleF inhibit and activate NF-κB signalling, respectively^32^. These dynamics obscure direct associations between individual effectors and infection outcomes, suggesting that effectors act within a robust and modular network. Consistent with this, we have recently demonstrated that effector networks can sustain extensive losses while maintaining colonisation, provided that a core set of essential and context-dependent effectors remains intact^21^. Only three effectors, Tir, NleA/EspI (affecting protein trafficking, tight junction assembly and cell death) and EspZ (the T3SS gatekeeper), are individually essential – deletion of any one significantly impairs in vivo colonisation^21^. The remaining effectors are conditionally essential, becoming critical only in specific combinations within the broader effector network. Several minimal effector networks have been characterised, each supporting colonisation but eliciting distinct host immune responses; for example, the strains CR_i9_ and CR_M12_ trigger hyper and hypo gut inflammatory states compared to inflammation induced by wild type CR (CR_WT_), respectively^21,33^.

Based on our previous work on T3SS effectors, we hypothesised that applying a rational attenuation strategy that targets non-essential effectors would enable the development of an LAV that balances attenuation and immunogenicity. Using CR as a model, we engineered a minimal effector network composed of only 10 effectors named CRV (CR Vaccine) that preserves colonisation but causes no detectable pathology in C57BL/6 mice. Immunisation with CRV resulted in protection against rechallenge with CR_WT_. This minimal effector network is maintained in clinical EPEC and EHEC strains, supporting its use as a rational attenuation strategy for the development of LAVs to protect against A/E pathogens.

## Results

### Constructing the CRV minimal network

**Fig. 1:**
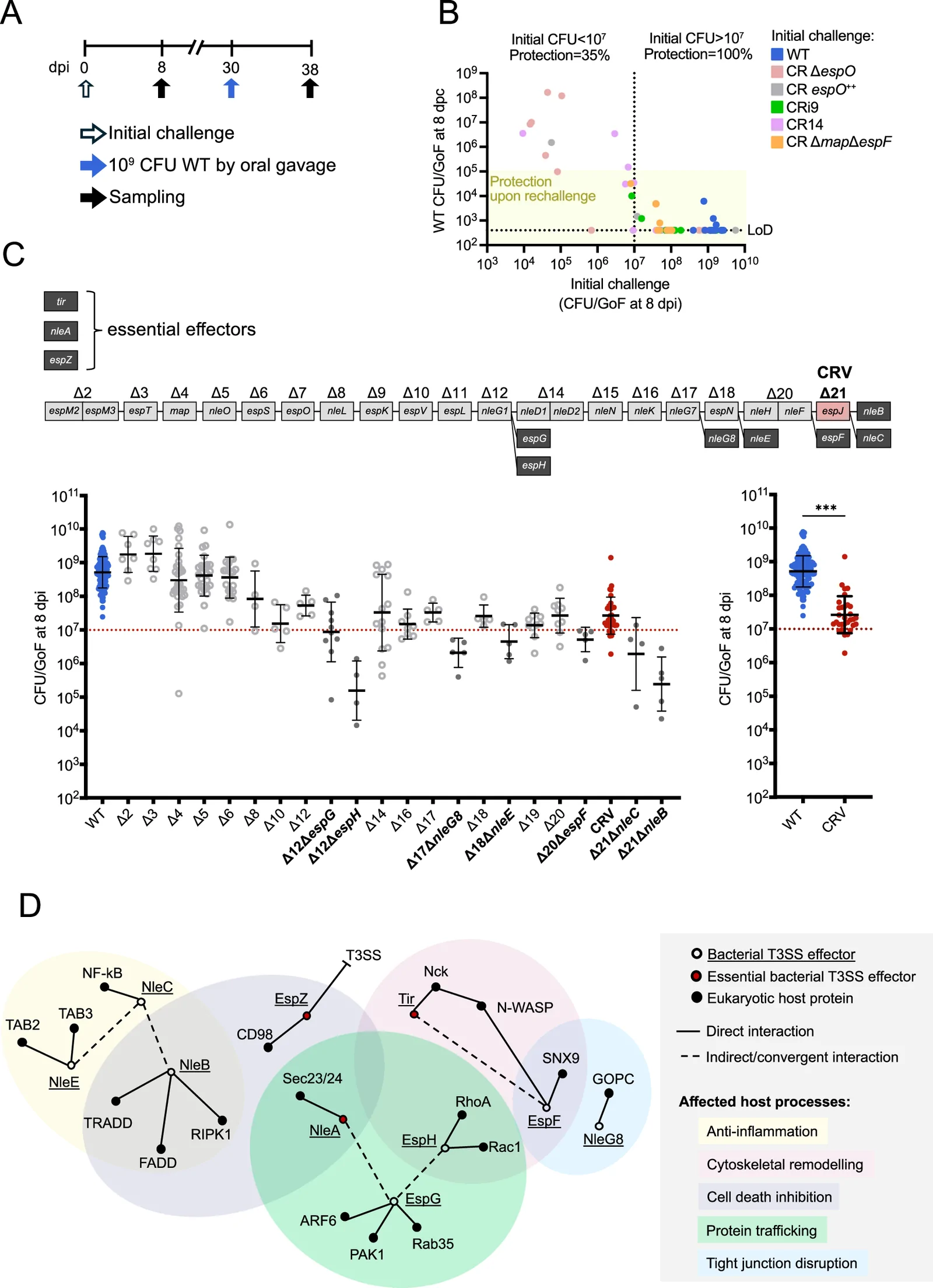
Generation of the CRV minimal effector network. **A** Experimental design of rechallenge experiments to determine the colonisation threshold necessary to induce protective immunity. **B** CR_WT_ CFU enumeration at 8 dpc versus bacterial burden during primary infection with CR_WT_ or CR effector mutants at 8 dpi. Each point represents the value of an individual mouse. The dotted line represents the limit of detection. **C** Non-essential T3SS effector deletion sequence and corresponding faecal CFU enumeration at 8 dpi. Those effectors which resulted in faecal shedding below the set threshold of 10^7^ CFUs/GoF were maintained within the network. The maintained effectors are represented by dark grey boxes. CRV was generated after the deletion of 21 effectors. The red dotted line represents the colonisation threshold. Line represents geometric mean ± geometric SD. Each data point represents one mouse (*n* = 24 (WT CR) and 7 (CRV) independent experiments). **D** Effectors forming the CRV network functionally converge on host processes to lower inflammation, inhibit cell death, remodel the cytoskeleton and disrupt tight junctions and protein trafficking. dpi = days post-infection, dpc = days post-challenge. LoC = limit of detection. Statistical difference was determined by a two-way Student’s *t* test; ***p < 0.001. Source data are provided as a Source Data file. Source Data

Reducing the effector repertoire is expected to compromise colonisation capacity, potentially imposing a trade-off between attenuation and protection. We therefore defined the level of colonisation required to confer protection. Utilising CR mutants lacking or over-expressing effector genes (e.g., Δ*espO*, over expression of *espO*, Δ*map/*Δ*espF*) or encoding minimal effector networks (e.g., CR_i9_ and CR_14_^21^) with distinct colonisation capacities, we identified a colonisation threshold that reliably predicted protection upon rechallenge. Strains reaching ≥10^7^ CFU/GoF at infection peak (8 dpi) conferred 100% protection against subsequent CR_WT_ challenge, defined as ≤10^5^ CFU/GoF at 8 days post-challenge (dpc) (Fig. 1A, B). In contrast, infections that colonised below the 10^7^ CFU/GoF threshold resulted in inconsistent protection (35%).

Guided by this threshold, we applied a systematic deletion strategy to target individual CR effector genes and construct a novel minimal effector network, utilising our mutagenesis vector library^21^. The essential effectors Tir, NleA/EspI and EspZ were excluded from the deletion pipeline. Genes were sequentially deleted and colonisation fitness assessed in C57BL/6 mice until bacterial faecal shedding at 8 dpi fell below 10^7^ CFU/GoF. Effector genes were retained only when their deletion reduced bacterial shedding below the established threshold. The deletion sequence was initiated with effector genes that modulate actin dynamics through Rho GTPase signalling – *espM2* and *espM3*, which activate RhoA, *espT*, which targets Rac1 and Cdc42, and *map,* which activates Cdc42^34,35^. From this point, deletions were non-hierarchical and guided by empirical evaluation of colonisation fitness (Fig. 1C).

The robustness of the network reached its limit following 21 effector deletions, with seven effectors – EspG, EspH, NleG8, NleE, EspF, NleC and NleB – proving to be conditionally essential to the network. The resulting strain, designated CRV (CR vaccine) and comprising only 10 effectors, colonised mice above the set threshold but at significantly lower levels than CR_WT_ (Fig. 1C). While many effector functions are undefined, all the retained effectors have assigned functions that converge on core host pathways to regulate cell death (NleA/EspI^34,35^, NleB^36,37^, EspZ^38,39^), inflammation (NleC^20^, NleB, NleE^40^), cytoskeletal remodelling (Tir^41,42^, EspF^19,43^, NleG8^44,45^, EspH^16^) and protein trafficking (NleA/EspI, EspG^46^, EspH) (Fig. 1D and Table 1). To ensure the reduced effector network did not impact A/E lesion formation, we assessed the ability of CRV to trigger actin polymerisation in vitro using the colonic mouse cell line CMT-93. CRV exhibited equivalent cell attachment and formation of actin-rich pedestal-like structures compared to CR_WT_. (Supplementary Fig. 1).

**Table 1:** The CRV effectors and their function

Effector	Activity	Partner proteins
Tir	Intimin receptor	Intimin, Nck
EspZ	T3SS gatekeeper	CD98
NleA	Inhibition of intracellular protein trafficking	Sec24
EspF	Disruption of tight junctions	SN9, N-WASP
NleG8	E3 ubiquitin ligase-like	
NleB	N-acetylglucosamine transferase of FADD-containing proteins	
NleC	Zinc-metalloprotease hydrolysing NF-kB RelA	
NleE	Cysteine methyltransferase of TAB2/3	
EspG	Affecting the recycling of host cell surface proteins	ARF6, Rab35, PAK1/2/3, GM130
EspH	Inhibits RhoGEF	Rab GTPases

### CRV causes minimal intestinal damage

**Fig. 2:**
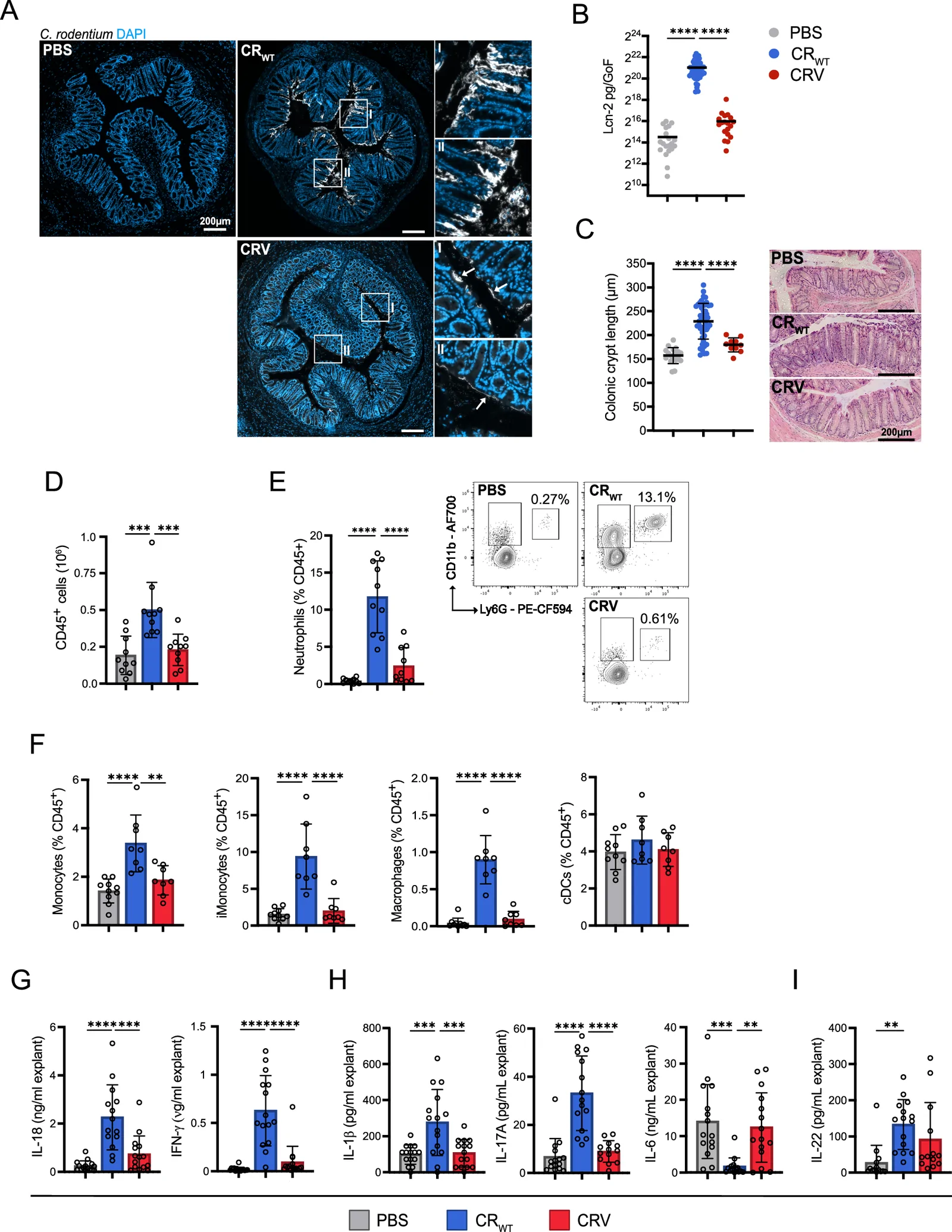
CRV causes minimal damage to the colonic mucosa at 8 dpi in C57BL/6 mice. **A** Representative images of infected distal colons from PBS-treated, CR_WT_- and CRV-infected mice, stained with polyclonal anti-CR antibodies (white) and cell nuclei (DAPI, blue). Arrows point to faint CRV staining. Scale bar = 200 μm. **B** Faecal LCN-2 levels determined from stool homogenate. Each dot represents an individual mouse value. Line represents mean, *n* = 5 (PBS), 15 (WT), and 4 (CRV) biological replicates, 2-5 mice per group. The line indicates the mean. **C** Colonic crypt length and representative H&E-stained images of the distal colonic section. Each dot represents the mean crypt length per mouse. Line indicates mean ± SD. Scale bar = 200 μm. *n* = 4 (PBS), 10 (WT CR) and 2 (CRV) biological replicates, 3-5 mice per group. **D**–**F** Colonic immune cell recruitment and sample dot plot (**E**) of neutrophil populations between treatment groups, and local cytokine levels (**G**–**I**). Each dot represents the values of an individual mouse. Lines represent mean ± SD. *n* = 2 (immune cells) or 3 (explant cytokine) biological replicates, 4-5 mice per group. Statistical significance was determined by one-way ANOVA with Tukey’s multiple comparison test, including all pairwise comparisons. Only significant comparisons are shown, non-significant comparisons (*p* > 0.05) are omitted; *p < 0.05; **p < 0.01; *** *p* < 0.001; ****p < 0.0001. Source data are provided as a Source Data file. Source Data

To evaluate the impact of CRV infection in C57BL/6 mice in vivo, bacterial localisation was first assessed by immunofluorescent staining of distal colonic sections at 8 dpi. CR_WT_ staining was dense and uniform along the mucosal surface (Fig. 2A). In contrast, while the CRV signal was sporadic and faint, it confirmed that CRV retains distal colonic tropism (Fig. 2A). Faecal lipocalin-2 (LCN-2), an antimicrobial protein involved in nutritional immunity and a marker of mucosal inflammation^47^, was elevated during CR_WT_ infection but decreased along the effector deletion sequence and remained at baseline in CRV-infected mice (Fig. 2B and Supplementary Fig. 2A). Histological analysis revealed a stepwise reduction in CCH along the deletion series, with crypt lengths reduced after the deletion of the first six effectors (EspM2, EspM3, EspT, Map, NleO and EspS) (Supplementary Fig. 2C). In CRV-infected mice, crypt length was indistinguishable from PBS-treated controls and significantly shorter than in CR_WT_-infected mice (Fig. 2C).

We next quantified infiltrating colonic immune cells in CRV-infected mice. This revealed comparable levels of cell recruitment to PBS-treated controls, with no increase in total CD45^+^ leukocytes within the lamina propria (Fig. 2D and Supplementary Fig. 2B). Neutrophil counts, key mediators of inflammation and tissue damage during CR_WT_ infection^48,49^, were also not significantly different to PBS treatment groups (Fig. 2E). Other myeloid subsets, including macrophages, monocytes, inflammatory monocytes and conventional dendritic cells, remained at baseline (Fig. 2F). T and B cell populations exhibited a similarly unchanged profile (Supplementary Fig. 2D). These cellular dynamics mirrored mucosal cytokine profiles. Pro-inflammatory cytokines generally upregulated during CR_WT_ infection, including interleukin (IL)-18, IFN-γ, IL-1β and IL-17A, remained at baseline in CRV-infected mice, equivalent to PBS controls (Fig. 2G, H). In contrast, cytokines that are suppressed during CR_WT_ infection, such as IL-6, IL-10 and the chemokine CCL3, were maintained upregulated in CRV infection (Fig. 2H and Supplementary Fig. 2E). The only exception was seen in IL-22 levels, which were intermediate between the PBS and CR_WT_ groups (Fig. 2I). This may reflect a mild level of epithelial stress sufficient to induce IL-22 release, which safeguards barrier integrity during CRV infection.

Attenuation was further evaluated by challenging C3H/HeN mice with CRV, alongside CR_WT_ as a control. This mouse strain is susceptible to CR_WT_ infection, which causes severe diarrhoea and dehydration^50^. At infection peak (6 dpi)^51^, CRV colonisation reached ~ 10^8^ CFU/GoF, significantly lower than shedding levels observed in CR_WT_-infected mice (Supplementary Fig. 3A). Importantly, CRV infection caused no mortality, and mice gained weight throughout the infection course (Supplementary Fig. 3B, C). In contrast, all CR_WT_-infected mice developed rapid weight loss, reaching humane endpoint by 12 dpi. Together, these data suggest that the CRV minimal effector network enables epithelial colonisation without eliciting overt tissue damage or immunopathology in resistant mice and is tolerated in susceptible C3H/HeN mice.

### CRV confers protection against subsequent CR_WT_ challenge

**Fig. 3:**
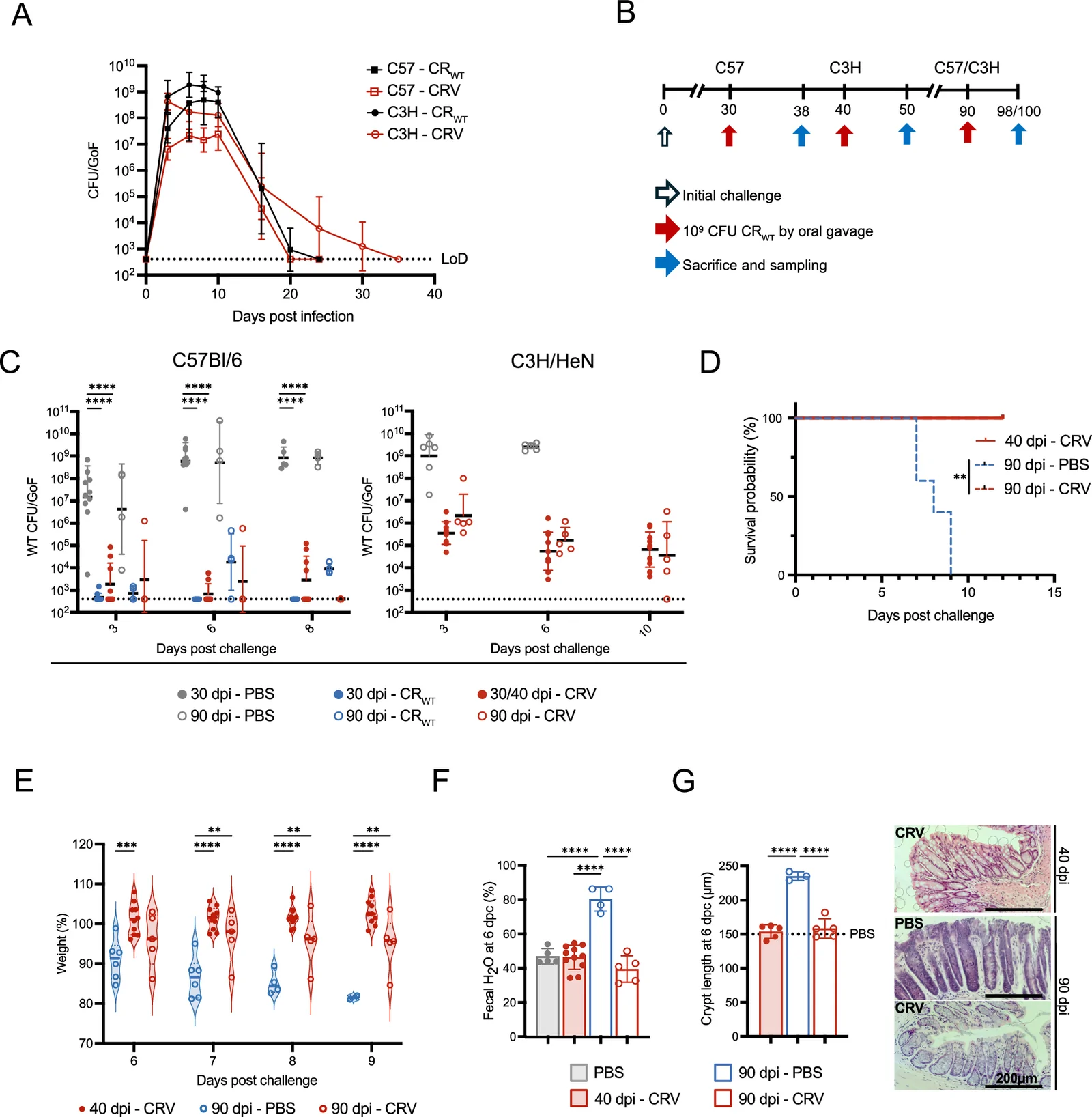
CRV prevents future infection and disease. **A** CR_WT_ vs CRV differential infection kinetics in C57BL/6 and C3H/HeN mice. *n* = 3 biological replicates, 4-5 mice per group. Dot represents geometric mean ± geometric SD. **B** Mice were initially challenged with either PBS, CR_WT_ or CRV and then subsequently rechallenged with CR_WT_ at either 30 (C57BL/6) or 40 (C3H/HeN) dpi and then at a late 90 dpi time point. **C** CFU/GoF at 3, 6 and 8 (C57BL/6) or 10 (C3H/HeN) dpc at both 30/40 and 90 dpi. Each dot represents the values of an individual mouse. Line is geometric mean ± geometric SD. *n* = 2 biological replicates for early time points (30/40 dpi), 1 biological replicate for 90 dpi. 3-5 mice per group. **D** Survival probability curve demonstrating that C3H/HeN mice survive WT challenge after vaccination with CRV. **E** C3H/HeN weight loss, (**F**) faecal water content and (**G**) CCH with representative images of H&E-stained colonic sections at humane endpoint (PBS-WT) or 10 dpc (CRV-WT). *n* = 1 biological replicate for late time points (90 dpi) and 2 for early time points (40 dpi). Lines indicate mean ± SD. dpc = days post challenge; dpi = days post infection, LoD = limit of detection. Statistical analysis was determined by calculating Area Under the Curve (**A**) followed by one-way ANOVA with Tukey’s multiple comparison test (**F**,** G**), log transformation (**C**) and two-way ANOVA with Bonferroni correction (**C**, **E**), and log-rank (Mantel-Cos) test (**D**), including all pairwise comparisons. Only significant comparisons are shown, non-significant comparisons (*p* > 0.05) are omitted; *p < 0.05; **p < 0.01; *** *p *< 0.001; ****p < 0.0001. Source data are provided as a Source Data file. Source Data

CR_WT_ infection results in protective immunity through humoral responses^29,30^. To confirm CRV infection induces a similar response, mice were rechallenged with CR_WT_ at either 30 (C57BL/6) or 40 (C3H/HeN) dpi, ~ 10 days after respective primary infection clearance. These timepoints accounted for the differing durations of primary infection between the mouse strains (Fig. 3A). Mice were also rechallenged at 90 dpi to evaluate the duration of the protective response (Fig. 3B).

CRV infection conferred protection against CR_WT_ rechallenge at both early and late timepoints in C57BL/6 and C3H/HeN mice (Fig. 3C). C57BL/6 mice exhibited minimal to no faecal bacterial shedding at 3-, 6- and 8-dpc, comparable to CR_WT_ - CR_WT_ rechallenge controls. In C3H/HeN mice, a reduced but detectable bacterial load (~ 10^5^ CFU/GoF) persisted. To confirm this did not reflect disease, weight trajectories were tracked and remained stable, with no mouse mortality recorded by 10 dpc (Fig. 3D, E). Faecal water content, a measure of diarrhoea, and CCH also remained comparable to PBS controls (Fig. 3F, G). These data demonstrate that CRV elicits a robust protective immune response that prevents disease following CR_WT_ rechallenge.

### Intimate attachment is required for protective immunity

**Fig. 4:**
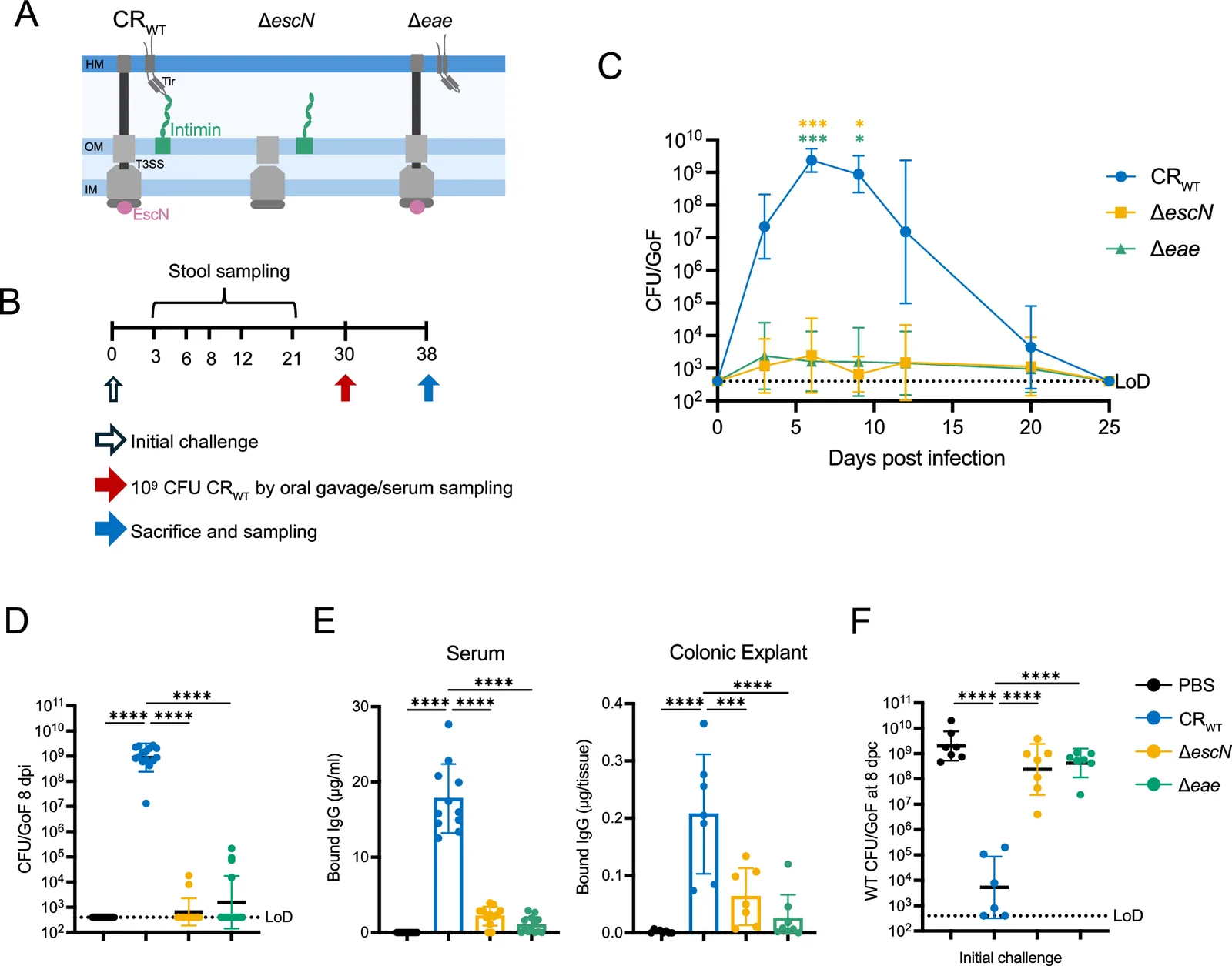
Severe attenuation does not induce protective immunity in C57BL/6 mice. **A** T3SS assembly and effector delivery and A/E lesion formation are ablated in Δ*escN* and Δ*eae* mutants, respectively. **B** Vaccine strategy schematic. Mice were challenged with respective treatments, the infection tracked, and then mice were rechallenged with CR_WT_ at 30 dpi to assess protective responses from the initial challenge. **C**,** D** Full infection kinetics of primary infection and CFU/GoF at 8 dpi. *n *= three biological replicates, 5 mice per group. Dot represents geometric mean ± geometric SD (**C**). Each dot represents an individual mouse value. Line represents geometric mean ± geometric SD (**D**). **E** Local and systemic IgG titres targeting CR, determined by heat-killed whole-bacterial cell ELISA. Each dot represents an individual mouse value, line represents mean ± SD. *n* = 3 (serum) or 2 (colonic explant) biological replicates, 3-5 mice per group. **F** CFU counts at 8 days post CR_WT_ rechallenge. *n* = 2, dpc = days post challenge, dpi = days post infection, LoD = limit of detection. Statistical difference was determined by calculating Area Under the Curve (**C**) or log transformation (**D** and **F**) and by one-way ANOVA (**C**–**F**) with Tukey’s multiple comparison test, including all _**T**_ pairwise comparisons. Only significant comparisons are shown, non-significant comparisons (p > 0.05) are omitted; **p < 0.01; *** *p* < 0.001; ****p < 0.0001. Source data are provided as a Source Data file. Source Data

To further validate the rationale of targeting non-essential effectors over essential metabolic or virulence genes that abrogate colonisation and replication, we assessed the protective potential of severely attenuated strains. To this end, two mutants were evaluated: Δ*escN*, lacking the ATPase that powers the T3SS and enables effector secretion, and Δ*eae*, deficient in intimin; both mutants are unable to intimately attach to the epithelium (Fig. 4A). C57BL/6 mice were infected with these mutants alongside PBS and CR_WT_ groups and rechallenged with CR_WT_ at 30 dpi (Fig. 4B). During primary infection, both Δ*escN* and Δ*eae* exhibited minimal colonisation, with either undetectable or significantly reduced faecal bacterial shedding throughout infection, compared to CR_WT_ (Fig. 4C, D). CR-specific IgG titres induced by both mutants were not significantly different from PBS controls and failed to confer protection, as mice shed high bacterial loads upon CR_WT_ rechallenge at 8 dpc (Fig. 4E, F). These findings, which are consistent with the set colonisation threshold (Fig. 1B), support an attenuation strategy that balances efficient colonisation and effector delivery while limiting host pathology. Moreover, they underscore the requirement for active effector delivery and intimate bacterial attachment to induce a protective immune response.

### Mapping the dominant CR antigens

**Fig. 5:**
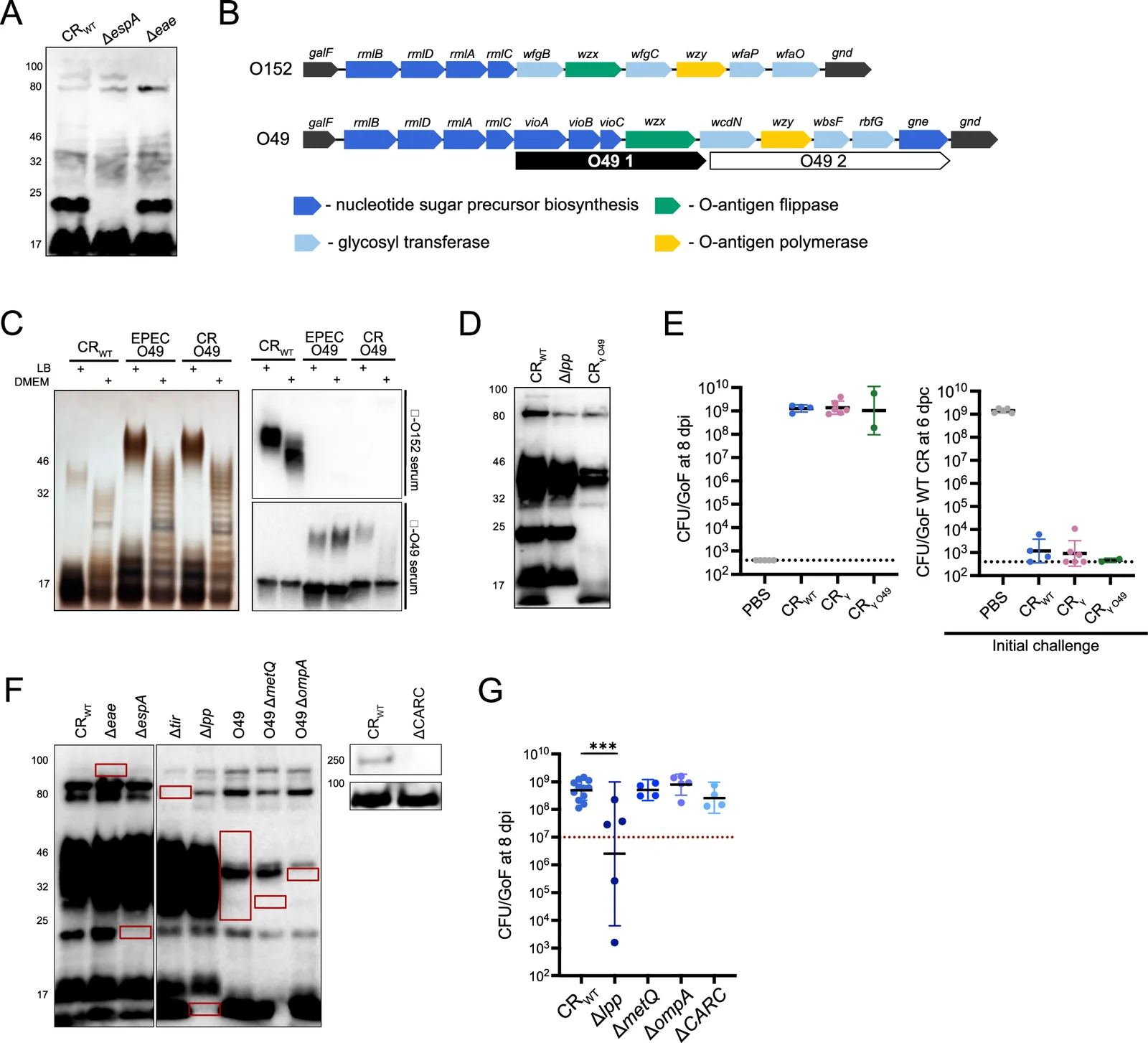
CR_WT_ and CRV infection induces antibodies against a conserved set of antigens. **A** An example WB demonstrating reactivity of convalescent serum from mice at 30 dpi to the Ler-regulated proteins intimin (~ 90 kDa) and EspA (~ 25 kDa). **B** O-antigen gene clusters of the O152 antigen, natively expressed by CR_WT_, and the O49 antigen. Switching of the gene clusters was performed in two cloning steps (O49 1 and O49 2) and preserved the genes *rmlB rmlD*, *rmlA* and *rmlC* which are shared by both loci. **C** Confirmation of O-antigen unit change from O152 to O49 by both Silver stain of extracted LPS and WB using anti-O152 and anti-O49 serum against whole bacterial lysates. **D** Representative WB image demonstrating convalescent serum from CR_WT_ infected mice is not reactive to γ variants of intimin and EspA or the O49 antigen expressed by CR_γ O49_. **E** Enumeration of CFU/GoF at 8 dpi and 6 dpc with CR_WT_. Each dot represents an individual mouse value. Line represents geometric mean ± geometric SD. *n* = 1, 2-6 mice per treatment group. **F** Example WB of individual antigen deletion mutant lysates probed with convalescent serum pooled from WT-infected mice at 30 dpi. **G** CFUs from mice infected with individual antigen deletion mutants demonstrating OmpA, MetQ and CARC do not contribute to bacterial virulence. Each dot represents values from an individual mouse. Line represents geometric mean ± geometric SD. The red dotted line represents the colonisation threshold. *n* = 3 (WT) or 1 (Δ*lpp,* Δ*metQ,* Δ*ompA,* Δ*CARC*) biological replicate, 4-5 mice per group. Statistical differences were determined by one-way ANOVA with Tukey’s multiple comparison test, including all pairwise comparisons. Only significant comparisons are shown, non-significant comparisons (*p* > 0.05) are omitted; *** *p* < 0.001. All blots are representative of at least four independent experiments utilising mouse serum pooled from distinct experimental groups, as well as from serum collected from individual mice. Source data are provided as a Source Data file. Source Data

Given that clearance of CR infection is mediated by IgG, we defined the dominant antibody targets and assessed their conservation following CRV vaccination. Previous reports identified the Ler-regulated proteins intimin and EspA (the filament that extends the T3SS needle) as dominant antigens targeted during CR_WT_ infection^31^. Western blot (WB) analysis using convalescent serum collected from CR_WT_-infected mice at 30 dpi against bacterial lysates confirmed strong antibody reactivity against both proteins but also revealed a broader antigenic profile (Fig. 5A). Based on a smeared banding pattern ~ 30–45 kDa, we hypothesised that antibodies also targeted the O-antigen component of lipopolysaccharide (LPS). Probing WB membranes containing purified CR LPS with convalescent serum confirmed reactivity against the O152 serotype express by CR_WT_ grown in either LB or DMEM (Supplementary Fig. 4A). In addition, a prominent ~ 18 kDa band was consistently observed in convalescent serum blots; based on previous reports of commensal-induced antibody responses^25^, this was suspected to be murein lipoprotein (Lpp), which is highly abundant and anchors the underlying peptidoglycan to the inner leaflet of the outer membrane^52^. Validation of its identity was confirmed by gene deletion and subsequent immunoblotting (Supplementary Fig. 4B).

Intimin, EspA and the O-antigen exist in multiple antigenically dissimilar isoforms across circulating EPEC and EHEC clinical isolates, presenting a potential barrier to cross-protection^53,54^. We therefore aimed to evaluate the presence of conserved antigens. To this end, we generated a CR strain expressing the EHEC isoforms of intimin and EspA (γ variants instead of the native β isoforms), termed CRγ. In addition, to bypass O-antigen recognition, the O152 antigen gene cluster was replaced with the O49 locus derived from a clinical EPEC isolate^55^. Given the large size of the gene cluster, the switch was performed in two steps, retaining the genes *rmlA*-*D* encoding rhamnose biosynthetic enzymes common to both O152 and O49 (Fig. 5B). Successful O-antigen replacement was confirmed by silver staining and serological analysis; CR_O49_ exhibited an LPS banding pattern identical to EPEC O49 by silver stain, lacked reactivity to anti-O152 serum, and was specifically recognised by anti-O49 serum (Fig. 5C). Consistent with this, anti-O152 and anti-O49 sera selectively agglutinated CR_WT_ and CR_O49_, respectively (Supplementary Fig. 4C). Moreover, convalescent sera from CR_WT_-infected mice did not recognise the γ variants of EspA and intimin or the O49 antigen, indicating a lack of cross-reactivity (Fig. 5D). Both CR_γ_ and CR_γ O49_ colonised mice similarly to CR_WT_ and infection with both strains provided full protection upon CR_WT_ re-challenge (Fig. 5E). These findings indicate that immune protection is not dependent on these antigens and instead involved additional protective antigens.

To identify the additional uncharacterised surface antigens recognised by IgG, we used a CR Δ*wfaO* mutant, which lacks a glycosyltransferase within the O152 gene cluster and generates rough LPS, to unmask antigens obscured by dominant O-antigen reactivity by WB. To determine if any antigens are regulated by the master LEE regulator Ler, we also assessed a Δ*ler* mutant and a Δ*ler/*Δ*wfaO* double mutant. WB analysis of theses strains revealed three additional reactive bands (~ 35, 40 and 80 kDa), with the antigen ~ 80 kDa found to be Ler-regulated (Supplementary Fig. 4D). Subcellular fractionation and membrane enrichment identified an additional high-molecular weight antigen (~ 250 kDa) and localised two of the other antigens to the outer membrane (~ 35 and 40 kDa) (Supplementary Fig. 4E). Mass spectrometry analysis determined the probable identity of these proteins as MetQ (methionine transporter lipoprotein - ~ 35 kDa), OmpA (a highly expressed porin - ~ 40 kDa) and CARC (AIDA-like autotransporter - ~ 250 kDa) (Supplementary Data 1), which were subsequently confirmed with deletion mutants. The Ler regulated band (~ 80 kDa) was identified as Tir, based on its molecular weight and exclusion from membrane fractions.

These findings define a core antigenic set comprising the O-antigen, virulence factors (intimin, EspA and Tir) and the outer membrane proteins Lpp, OmpA, MetQ and CARC) (Fig. 5F). While band intensity between individual mouse sera varied, all tested antisera were reactive to this core antigenic set. Of the none-virulence proteins, only Lpp was essential for mouse colonisation – all other single antigen deletions resulted in no change to colonisation (Fig. 5G).

**Fig. 6:**
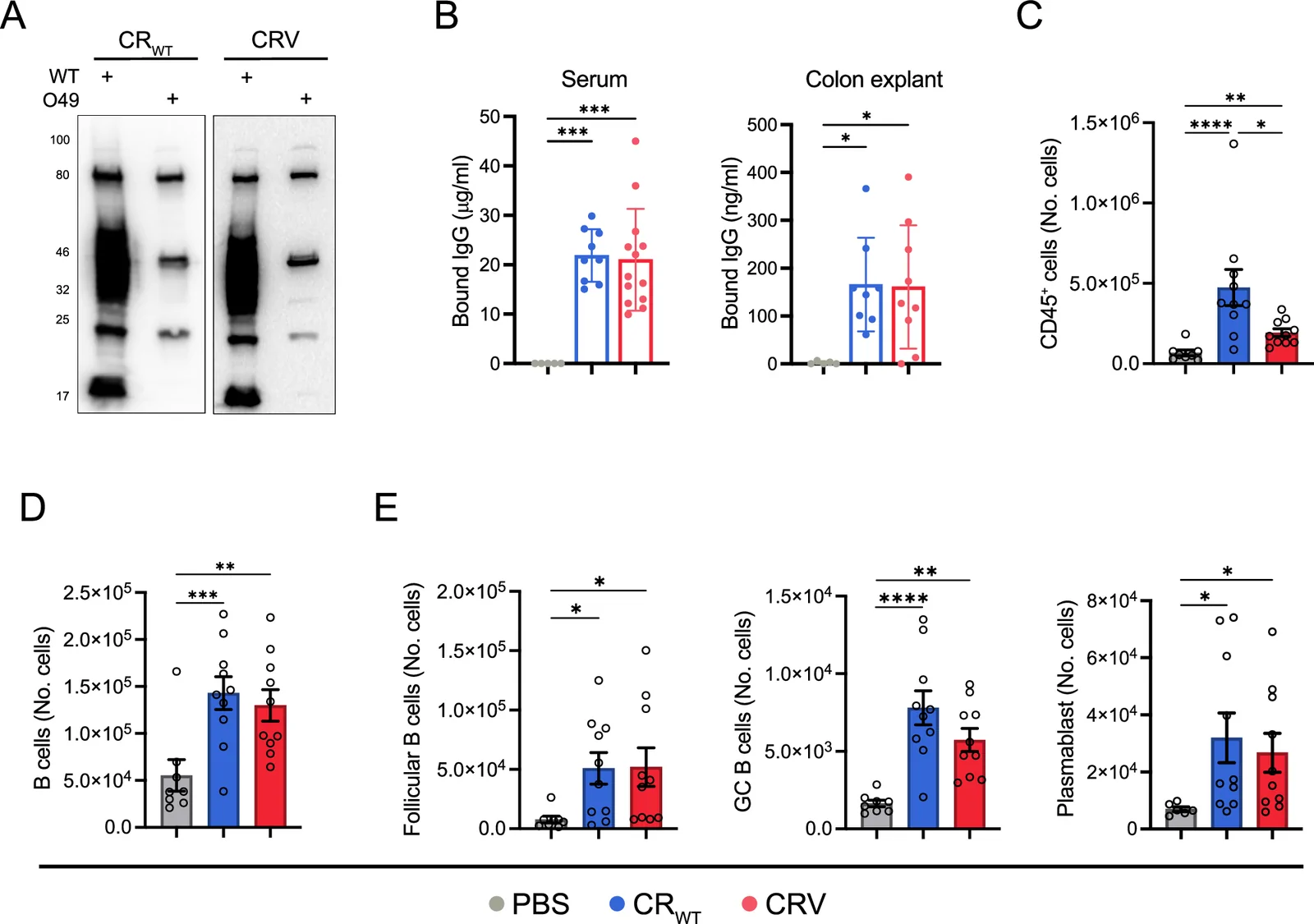
CRV and CR_WT_ infection elicit comparable antigen specificities and lamina propria B cell responses. **A** Example WB of antigenic profile from serum collected from CR_WT_- or CRV-infected mice at 30 dpi, pooled from individual experiments. Serum from individual mice shows the same qualitative antigen recognition pattern. Example blot from two independent experiments. **B** CR-specific serum and colonic explant IgG titres from PBS, CR_WT_- and CRV-infected mice at 30 dpi. Each dot represents an individual mouse value. Line represents mean ± SD. *n *= one (PBS) or three biological replicates for serum and two biological replicates for colon explants, 4-5 mice per group. Number of CD45^+^ cells (**C**), B cells (**D**) and B cell subsets (GC B cells, follicular B cells, and plasma blasts) (**E**) at 30 dpi. Each dot represents a value from an individual mouse. Line represents mean ± SD. *n *= two biological replicates, 4 (PBS) or 5 mice per group. dpi = days post infection. Statistical differences were determined by one-way ANOVA with Tukey’s multiple comparison test, including all pairwise comparisons. Only significant comparisons are shown, non-significant comparisons (*p* > 0.05) are omitted; *p < 0.05; **p < 0.01; *** *p* < 0.001; ****p < 0.0001. Source data are provided as a Source Data file. Source Data

Finally, CRV immunisation induced antigen recognition similar to CR_WT_ infection. Convalescent sera from CRV- and CR_WT_-infected mice showed reactivity against the same core antigenic set, and circulating and local IgG titres were equivalent between both groups at 30 dpi (Fig. 6A, B). Likewise, CD45^+^ cell infiltrates in the colonic lamina propria were significantly reduced in CRV-infected mice compared to CR_WT_ infection at 30 dpi, but CRV infection induced comparable numbers of local B cell populations (Fig. 6C, D and Supplementary Fig. 4). This included germinal centre (GC) B cells, follicular B cells, and plasma blasts (Fig. 6E), indicating that CRV maintains robust humoral priming despite limiting pathology.

### EPEC expressing the CRV effector network colonises IECs in a gut-on-chip model

**Fig. 7:**
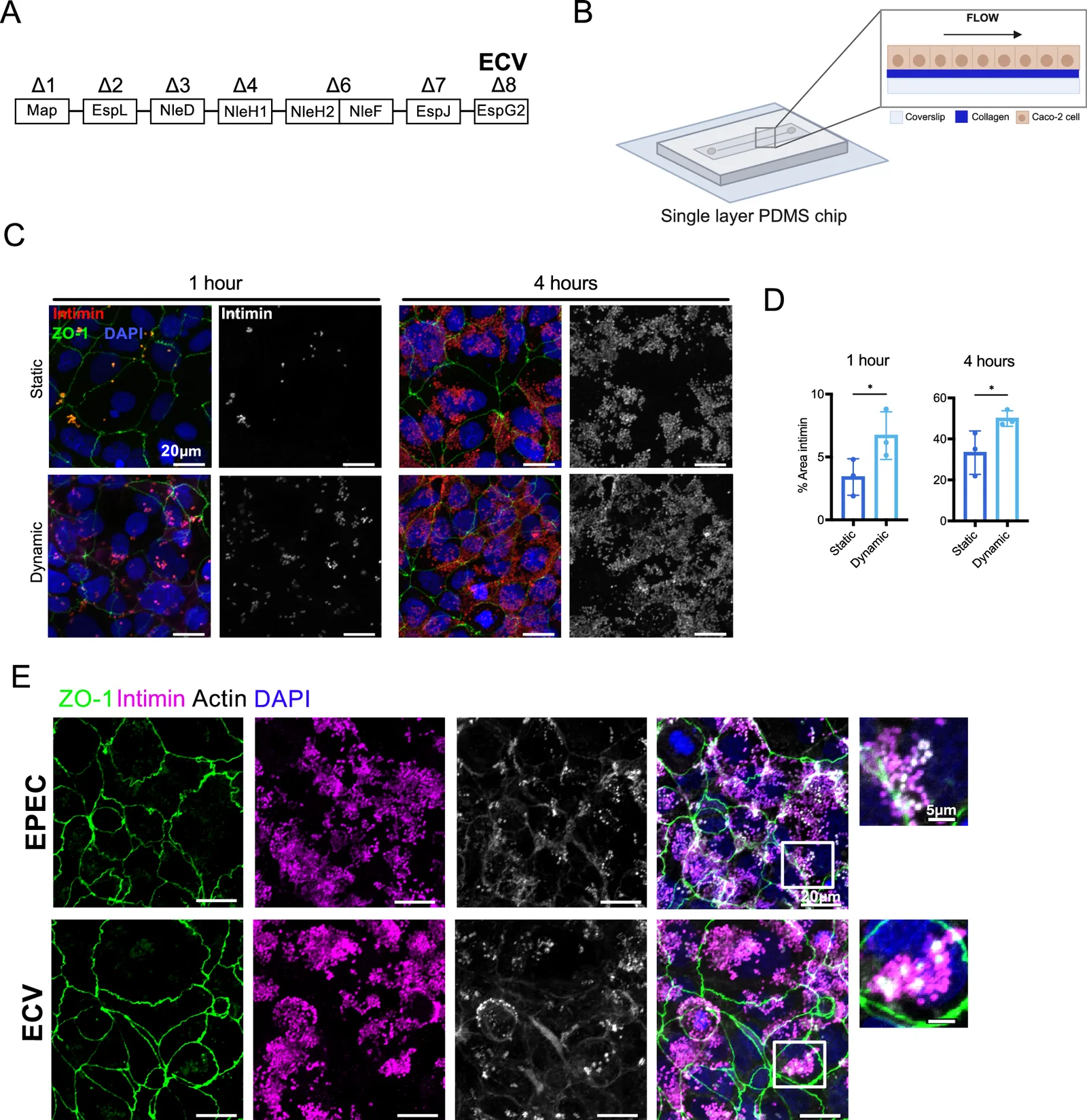
The EPEC vaccine (ECV) strain colonises Gut-on-Chip IECs . **A** Accessory effector deletion sequence in EPEC to create ECV. **B** Gut-on-Chip (GoC) design, cells were grown on collagen-coated microfluidic chips, which allow media to flow dynamically through the chamber at physiological rates (60 μl/h). **C** Representative images of GoC EPEC infection of Caco-2 cells grown either statically or dynamically. Images show nuclei (DAPI), tight junctions (ZO-1, green), and adherent bacteria (intimin, red). *n* = 3 biological replicates. **D** Quantification of bacterial binding. Each dot represents mean ± SD from three images from one biological experiment. **E** EPEC and ECV infection of Caco-2 cells grown dynamically on the GoC model. Images show nuclei (DAPI), tight junctions (ZO-1, green), and adherent bacteria (intimin, red) and actin-rich pedestal formation (pink). Images are representative from 3 independent experiments. Statistical differences were determined by a one-tailed Student’s *t* test. Tukey’s multiple comparison test; *p < 0.05. Source data are provided as a Source Data file. Source Data

Despite variation in effector repertoires across A/E pathogens, the CRV effector network is conserved in clinical EPEC and EHEC strains, including the prototypical EPEC strain E2348/69^56,57^. To evaluate whether this effector network is sufficient to support EPEC epithelial colonisation, we generated an EPEC equivalent of CRV by sequentially deleting the accessory effectors, resulting in a strain we named ECV (EPEC vaccine) (Fig. 7A). To establish whether ECV would colonise cells in vitro, we optimised a gut-on-chip (GoC) infection model using Caco-2 cells grown with media either statically or flowing dynamically across a microfluid chip (Fig. 7B). We found that EPEC was more adherent to cells grown in a dynamic system, perhaps due to increased surface area (Fig. 7C, D). We next infected the GoC model with either EPEC or ECV and found that epithelial adherence and pedestal formation were equivalent between the treatment groups (Fig. 7E), confirming that the CRV/ECV network supports colonisation and Tir-induced actin polymerisation both in vivo and in vitro.

Together, we have shown that a minimal effector network of ten effectors is sufficient to sustain epithelial colonisation and define a tractable framework for live attenuation and vaccine design against A/E pathogens.

## Discussion

Live attenuation has largely been supplanted by modern vaccine platforms, including subunit, mRNA and conjugate technologies. However, for mucosal bacterial pathogens, live-attenuated and inactivated whole-cell vaccines remain the few licensed options. Subunit-based vaccines are poorly immunogenic at mucosal surfaces, prone to degradation and currently lack effective adjuvants capable of inducing protection at the site of infection^1^. Classical attenuation technologies have yielded successes, notably *Salmonella* Typhi Ty21a (Vivotif), developed through chemical mutagenesis, which gives protection following oral ingestion of 4 doses^58,59^. More targeted strategies, such as auxotrophic mutants, for example, the *Salmonella* Δ*aroC*/*ssaV* strain lacking aromatic amino acid biosynthesis and the T3SS-2 ATPase, may elicit antibody responses but fail to protect against infection^60^. To date, *Vibrio cholerae* CVD 103-HgR (Vaxchora), which lacks the ADP-ribosylating A subunit of the cholera toxin, remains the only licensed example^61,62^. Increased understanding of bacterial pathogenesis could enable rational attenuation methods that retain key host-pathogen interactions while preventing disease.

To explore this approach in the context of A/E pathogens, we engineered an attenuated CR strain by contracting its T3SS effector network. The CRV network, comprising the effectors Tir, NleA, EspZ, EspG, EspH, NleC, NleB, NleE, EspF, and NleG8, is sufficient for colonisation but limits overt tissue disruption. This set preserves critical functions involved in immune modulation, epithelial engagement and cytoskeletal remodelling. For example, the retained effectors NleC and NleE both converge on NF-κB inhibition. These effectors dampen the transcription of proinflammatory cytokines, reflected in both infiltrating immune cell populations and local cytokine secretion in CRV-infected mice. Previous studies have shown that the activity of the anti-inflammatory effectors must be functionally counterbalanced by effectors that inhibit cell-death, including NleB, NleF or EspL; here, NleB appears sufficient to stabilise this balance^21^. Importantly, ECV, comprising the same effector set, efficiently colonised IECs in a gut-on-chip model. Although this system captures only early interactions and does not reflect the full complexity of the gut environment, it indicates that deletion of accessory effectors does not impair epithelial attachment by EPEC.

Many of the effectors excluded from the CRV network represent accessory nodes previously shown to be dispensable in other minimal effector networks, namely Map, NleD1/2, NleH. NleF, EspJ, NleG1, NleG7, EspV, EspK, NleN, NleK. While not essential to colonisation, these effectors may confer selective advantages in vivo, fine-tune disease severity or optimise transmission^33^. Their removal from CRV highlights not only a rational path to attenuation but also contributes toward defining the minimal genetic circuitry required to sustain infection.

Among the local cytokines measured, only IL-22 was elevated above baseline in CRV-infected mice. This response is likely driven by the retention of EspF within the CRV network. EspF disrupts mitochondrial function and tight junction integrity^63^, and IL-22 is required to repair the epithelial damage; *Il22*^−/−^ mice, which generally succumb to infection with CR_WT_, survive infection with Δ*espF*^19^. Although EspF is strongly associated with epithelial damage, its context within the reduced CRV network, and absence of other TJ-targeting effectors such as Map, appears to promote a beneficial cytokine response that safeguards the integrity of the mucosal barrier.

The network design is coupled with an inherent safety advantage, as deletion of multiple genes which are dispersed around the bacterial chromosome significantly reduces the likelihood of reversion. However, the generalisability of this minimal network is constrained by the host context in which it was defined. CRV was generated and validated in C57BL/6 mice that were age-matched, with minimal variations in host genetics or microbiota composition. While this provides experimental consistency, it also represents a limitation, as the microbiota is a key determinant of colonisation resistance and immune priming. Differences in microbiota composition could alter host defences and niche competition, thereby shifting the set of accessory effectors required to sustain infection and induce protection. Adaptations of effector composition may be required in hosts with distinct microbiomes, including other animal species, ruminants and humans.

Protection against CR infection required intimate interaction between the bacterium and the underlying intestinal epithelium. Oral immunisation with either Δ*escN* or Δ*eae* mutants induced CR-specific IgG responses not significantly different than mock infected mice, which were insufficient to confer protection against a future CR_WT_ challenge. Consistently, protection has been shown to be reliant on induction of local intestinal immunity, as intraperitoneal inoculation of heat-killed CR or subunit vaccines do not protect against subsequent challenge^64^.

In contrast, immunisation of rabbits with rEPEC expressing truncated intimin (lacking the carboxy-terminal Tir-binding domain) conferred protection against a homologous challenge while failing to protect against different serotypes and isolates^65,66^. An EHEC Δ*ler*/*stx2* strain complemented with plasmid-encoded detoxified Stx provided partial protection (55–70%) in a mouse model, although this required multiple vaccine doses delivered intraperitoneally prior to challenge^67^. Our findings suggest that CRV retains the ability to stimulate protective immunity through mucosal engagement via a natural infection route, though further work is required to evaluate efficacy across diverse serotypes.

Tir, intimin and EspA are well-defined antigens targeted during A/E pathogen clearance. Antibodies from human sera, colostrum and breast milk from mothers in regions where EPEC infection is endemic demonstrate reactivity to all three proteins^68–70^ and can inhibit bacterial adherence to HeLa cells in vitro^71^. Moreover, bovine colostrum contains IgG antibodies against intimin and EspA that inhibit the haemolytic activity mediated by the T3SS^72^. Our serological analysis builds on these discoveries to reveal an expanded antigen repertoire that includes several conserved outer membrane proteins in addition to virulence factors and the O-antigen.

While the O-antigen elicited strong immunoreactivity, replacement with the O49 serotype did not impair protection following rechallenged with CR_WT_, indicating that additional antigens contribute to protective immunity. Several of the identified targets are known to be immunogenic during other bacterial infections and have established vaccine potential. For example, the lipoprotein MetQ is a leading subunit vaccine candidate for *Neisseria gonorrhoeae*^73,74^, and OmpA has been extensively explored for protection against *Acinetobacter baumannii*^75^. AIDA-like autotransporters such as CARC and its EPEC homologue belong to a family of antigens that includes licensed vaccine components like Pertactin, a key constituent of the acellular pertussis vaccine^76,77^. These autotransporters have also been used as scaffolds for the surface display of heterologous antigens^78^. Our findings, therefore, support the broader utility of this system not only for live attenuation against A/E pathogens but also as a potential vehicle for mucosal antigen display (e.g. ref. ^79^. While antigens whose epitopes are conformational may have been missed, this work expands the known antigenic repertoire associated with A/E pathogenesis and opens new avenues for antigen discovery and vaccine design.

T3SS are conserved across enteric pathogens infecting humans and animals, and while effectors can be homologous across species, they are deployed in pathotype-specific combinations that drive distinct disease mechanisms and host adaptation^80^. Beyond A/E pathogenesis, the effector network concept has been validated in *Salmonella enterica* serovar Typhimurium, where a reduced set of just six effectors from a total of thirty were sufficient to maintain pathogenesis in mice, with the transcriptional regulator SpvR required for full network functioning in vivo^81,82^. While effector networks have largely been used to dissect bacterial pathogenesis, their engineering could offer a framework for the design of LAVs in T3SS-utilising organisms, including WHO-prioritised pathogens such as *Salmonella*, *Shigella* and *Yersinia* spp.

Here, we demonstrate that effector network engineering in the context of CRV/ECV offers a path toward LAVs for EPEC and EHEC, pathogens for which safe and effective vaccines are needed to protect both humans and cattle and help alleviate the burden of diarrhoeal disease.

## Methods

### Ethical statement

All animal work took place at Imperial College London (Association for Assessment and Accreditation of Laboratory Animal Care accredited unit) under the auspices of the Animals (Scientific Procedures) Act (UK) 1986 (PP7392693). Work was approved locally by the institutional ethics committee (AWERB).

### Bacterial cultures

Bacterial strains used in this study are listed in Supplementary Data 2. Cultures were grown at 37 °C on Lysogeny broth agar (LBA) plates (1.5% w/v) or in liquid LB or Dulbecco’s modified Eagle’s medium (DMEM). When required, antibiotics were added as follows: nalidixic acid (Nal; 50 μg/ml), gentamicin (Gm; 10 μg/ml), and streptomycin (Sm; 50 μg/ml). For T3SS induction, CR and EPEC were cultured in 5 ml LB (8 h (h), 37 °C, 200 rpm), then sub-cultured into 5 ml DMEM and incubated overnight (o/n) statically at 37 °C and 5% CO_2_.

### DNA constructs and mutant generation

Primers and plasmids are listed in Supplementary Data 2. Bacterial gene deletions were performed using our effector mutagenesis vector library^21^. Deletions and gene replacements were generated using I-Sce/λ-Red recombination. For each target locus, approximately 500 bp homology regions (HR) located immediately upstream and downstream of the target gene were PCR-amplified using Q5 High-Fidelity 2X Master Mix (New England Biolabs (NEB)) or FantaFlash (Vazyme) and cloned into the SacI-SphI sites of pSEVA612S by Gibson Assembly (NEB). For deletions, the upstream and downstream HRs were joined directly to generate an in-frame scarless deletion upon recombination. For gene replacements, the desired coding sequence was PCR-amplified from the relevant genome and inserted between the HRs in pSEVA612S by Gibson Assembly, thereby placing the replacement sequence at the native chromosomal locus defined by the flanking HRs.

Constructs were transformed into *E. coli* CC118λpir and then conjugated into CR containing pACBSR via tri-parental conjugation, using *E. coli* 1047 pRK2013 as the helper strain. Conjugants were selected on LBA+Gm+Sm and then cultured in LB + Sm + 0.4% L-arabinose at 37 °C, 200 rpm to induce recombination. Cells were streaked on LBA+Sm and counter-selected with LBA + Gm. DNA constructs and gene deletions were confirmed by sequencing (Eurofins). Molecular cloning primers were designed using Benchling (www.benchling.com).

### Outer membrane preparation

Overnight cultures were subcultured in 10 ml of fresh media in 50 ml Falcon tubes and incubated at 37 °C for up to 3 h until an OD_600_ of between 0.6-1 was achieved. Cells were pelleted at 4000 RPM for 10 min at 4 °C and then washed in 1 mL PBS and again centrifuged for 5 min at 5000 x *g*. Cells were resuspended in 10 mM HEPES (Gibco), transferred to 2 ml Eppendorfs and centrifuged at 500 x *g* for 10 min at 4 °C. Pellets were resuspended in 1 ml HEPES and then sonicated at maximum amp (25%) for 15 cycles (10 s on, 20 s off) until solutions were clear. Samples were then centrifuged at 3000 x *g* for 10 min at 4 °C to pellet cellular debris. Supernatant was transferred to a new Eppendorf and centrifuged at 14000 x *g* for 30 min at 4 °C. Samples were then resuspended in 0.4 mL 10 mM HEPES with 2% sarcosine (Thermo Scientific) and incubated with light rolling for 30 min at RT. Samples were again centrifuged, as previously, and then resuspended in 50 ul of Mili-Q H_2_O. Protein concentrations were normalised before running on 12.5% SDS-PAGE gels (Bio-Rad).

### In vitro CR infection

CMT-93 cells were cultured in supplemented DMEM (10% FBS, 2 mM glutamine) at 37 °C and 5% CO_2_. For infection, cells were seeded at a density of 10^5^ cells/well on sterile glass coverslips. Overnight bacterial culture (50 ml) (multiplicity of infection = 50) was added to wells along with unsupplemented DMEM. Infections were synchronised by centrifugation at and then incubated for 4 h at 37 °C with 5% CO2. Infections were stopped by washing in PBS followed by fixation in 4% PFA. Following three PBS washes, samples were incubated in 0.1% Triton in PBS. Wash steps were repeated, and then samples were blocked in 3% BSA-PBS. Primary antibodies (anti-CR) were added in 1% BSA-PBS for 1 h. After washing, samples were incubated in secondary antibodies anti-rabbit IgG conjugated to Alexa647, with TRITC-conjugated phalloidin (1:100) and DAPI (1:1000) in 1% BSA-PBS for 45 min. Coverslips were mounted and imaged using a Zeiss AxioVision Z1 microscope (Zeiss).

### Mouse infections

All animal experiments complied with UK Home Office regulations and ARRIVE guidelines. The mouse work is covered by a Home Office project licence number PP7392693. Pathogen-free female C57BL/6 (6–8 weeks, 18–20 g) and C3H/HeN (8–10 weeks, 15–20 g) mice, purchased from Charles River Laboratories (UK). Only female mice were used as the CR model shows no reported sex-dependent differences in the assessed parameters. Sex was therefore not analysed as a variable. Mice were housed in high-efficiency particulate air (HEPA)-filtered cages with sterile bedding and environment enrichment, on a 12 h light/12 h dark cycle and ad libitum access to food and water. Animals were identified by ear notching. For each experiment, mice were age-matched and randomly assigned to experimental groups, with 4-5 mice per group and 2-3 independent biological replicates, unless otherwise stated within the figure legends. Mice were randomly assigned to cages on receipt. Allocation of cages to experimental groups was performed by the investigators and was not blinded.

For infection, CR cultures were grown o/n in 15 ml LB (37 °C, 200 rpm). Cultures were then pelleted and resuspended in 1.5 ml sterile PBS. Mice were orally gavaged with 200 μL of the suspension, containing 10^9^ CFUs, which was confirmed retrospectively by serial dilution and quantification of CFU growth on LBA + Nal. Colonisation was monitored by plating stool samples, as previously described^21^, at indicated time points. Mouse body weight was tracked throughout, and mice were euthanised upon reaching humane endpoint (> 20% weight loss).

### Blood collection and processing

For serum antibody analysis, blood samples were collected either by tail vein laceration (~ 20 μl per mouse) or via cardiac puncture. For the latter, mice were anaesthetised by intra-peritoneal injection of ketamine (100 mg/kg) and medetomidine (1 mg/kg) prior to sample collection. Blood was collected into BD Microtainer® tubes with Microgard™ closure with SST™ gel clot activator. Samples were left to clot and then centrifuged to isolate serum, which was stored at − 70 °C until processing.

For haematological analysis, 100 μl of whole blood was collected via cardiac puncture into potassium EDTA (K3 EDTA) tubes to prevent coagulation. Samples were analysed using a VETSCAN® HM5 Haematology Analyser (Zoetis) to quantify circulating immune cell populations.

### Histological analysis and immunostaining

Distal colon segments (0.5 cm) were fixed in 4% PFA for 2 h and then maintained in 70% ethanol. Samples were paraffin-embedded, sectioned at 5 μm and stained with hematoxylin and eosin (H&E) or processed for immunofluorescence. H&E-stained slides were used to determine hyperplasia by measuring ≥ 10 well-oriented crypts per mouse to calculate mean length.

For immunostaining, slides were dewaxed in Histo-Clear solution (VWR) twice for 10 min, and then serially in 100% ethanol (2x, 10 min), 95% ethanol (2x, 3 min), 80% ethanol (3 min) and PBS-0.1% Tween 20-0.1% saponin (PBS-TS) (2x, 3 min). Slides were then heated for 30 min in demasking solution (0.3% trisodium citrate-0.05% Tween 20 in distilled H_2_O) and then blocked in PBS-TS + 10% normal donkey serum (NDS) for 20 min in a humid chamber. Sections were incubated with primary antibody (anti-intimin, 1:50) in PBS-TS with 10% NDS for 60 min. Slides were then rinsed in PBS-TS (2 x, 10 min), followed by incubation with secondary antibodies (1:100) and DAPI (1:1000) (antibody details can be found in Supplementary Data 2). Wash steps were repeated, and slides were then mounted with ProLong Gold antifade mountant (Thermo Fisher Scientific). Images were acquired using a Zeiss AxioVision Z1 microscope, using an AxioCam MRm camera, and processed using Zen 2.3 Blue Version (Carl Zeiss MicroImaging GmbH, Germany).

### O152 and O49 antisera agglutination

Heat-killed agglutination was performed on the indicated strains after o/n growth in LB. Images were acquired on a ChemiDoc XRS + and visualised using Image Lab software (Bio-Rad Laboratories).

### ELISAs

For analysis of faecal samples, faecal pellets were homogenised in PBS-0.1% Tween 20 (PBS-T; 1 ml per 0.1 g faeces), centrifuged at 21,000 x *g* for 10 min, and supernatants stored at − 70 °C. Faecal LCN-2 levels were quantified using the DuoSet mouse LCN-2 ELISA (Bio-Techne) per the manufacturer’s instructions.

For serum and explant ELISAs, CR was grown o/n in DMEM to induce T3SS expression, normalised to an OD_600_ of 1 and heat killed in PBS with cOmplete™ Protease Inhibitor Cocktail (Roche) for 60 min at 60 °C. Samples were diluted 1:50 in carbonate coating buffer (0.1 M NaHCO_3_, 0.034 M Na_2_CO_3_ in dH_2_O), and 100 μl per well was used to coat high-binding 96-well Maxisorp plates o/n at 4 °C. For standard curves, wells were coated with goat anti-mouse IgG antibody (2 μg/ml). Plates were washed 3 times in PBS-T, then blocked in 3% bovine serum albumin (BSA)-PBS-T for 2 h at RT. Mouse serum and explant samples were added in duplicate (infected serum 1:2000, PBS controls and explants 1:500), alongside serial dilutions of purified mouse serum IgG as the standard and incubated o/n at 4 °C. After washing, plates were incubated in horseradish peroxidase (HRP)-conjugated goat anti-mouse IgG antibody (1:5000; Jackson Immunoresearch) for 60 min. Plates were developed by adding 3,3′,5,5′-tetramethylbenzidine (TMB; Thermo Scientific) and the reaction stopped with 2 N orthophosphoric acid. Absorbance was measured at 450 nm with 540 nm background correction using a FLUOstar Omega microplate reader (BMG Biotech). Anti-CR IgG levels were quantified by interpolation into to the IgG standard curve.

### Faecal water content

Faecal water content was determined from freshly collected faeces in pre-weighed 1.5 ml Eppendorfs. These were weighed again, the tube caps punctured, and then incubated at 55 °C for 48 h, after which no further decrease in weight was observed. Weight was again recorded, and faecal water content determined by the difference in weight between wet and dry faecal matter.

### Protein gels and western blot

Bacterial cultures were normalised to an OD_600_ of 1 in 1 ml, centrifuged at 4000 x *g* for 2 min. Bacterial pellets were resuspended in 60 μl of 2X Laemmli buffer (Bio-Rad), boiled at 95 °C for 10 min, and centrifuged at maximum speed for 2 min. Bacterial lysates were run on 12.5% SDS-PAGE gels (Bio-Rad).

Following electrophoresis, gels were either stained with Coomassie Brilliant Blue or transferred to 0.2 μm PVDF membranes (Thermo Scientific). Membranes were blocked in 5% skim milk in PBS-T for at least 2 h, then incubated o/n at 4 °C with convalescent mouse serum pooled from individual experimental groups, diluted 1:2000 in 5% BSA-PBS-T. Each immunoblot was independently repeated at least twice using serum from different biological replicates to ensure reproducibility. After 3 × 10 min washes in PBS-T, membranes were then incubated for 60 min at RT in HRP-conjugated goat anti-mouse IgG antibody (1:10,000, Jackson Laboratory). Following an additional three washes, the signal was developed using enhanced chemiluminescence substrate (GE Healthcare) and the mem. branes were imaged using the ChemiDoc Image Lab (Bio-Rad).

### LPS extraction and silver stain

O/n bacterial cultures were adjusted to an OD_600_ of 1 in 1 ml and pelleted. Cell pellets were resuspended in 1 x Laemmli buffer (Bio-Rad) and heated at 95 °C for 5 min. Proteinase K (20 mg/ml) (Qiagen) was then added to the samples, after which they were incubated for 2 h at 60 °C. Subsequently, 10 ul of 2-mercaptoethanol was added, and samples were boiled again for 10 min before centrifugation at maximum speed for 2 min. The supernatants were immediately loaded onto 12.5% SDS-PAGE gels (Bio-Rad) for separation.

Gels were first fixed o/n in 100 ml fixing solution (40% isopropanol, 5% acetic acid). Gels were then washed twice in 100 ml Mili-Q H_2_O for 5 min/wash and then soaked in periodic acid solution for 5 min to oxidise LPS (0.7% periodic acid, 40% isopropanol, 5% acetic acid made in Mili-Q H_2_O). After 2 x washes in Mili-Q H_2_O, steps were completed as per the SilverQuest™ Silver Staining Kit (Thermo Scientific) protocol and imaged using an iPhone 13.

### In-gel digestion and proteomics

Gel pieces were destained twice in 50 mM triethylammonium bicarbonate (TEAB) containing 50% acetonitrile (ACN) for 30 min each with gentle agitation, dehydrated in 100% ACN, and air-dried. Disulfide bonds were reduced with 5 mM tris(2-carboxyethyl)phosphine (TCEP) in 50 mM TEAB for 45 min at room temperature, followed by alkylation with 50 mM iodoacetamide (IAA) in 50 mM TEAB for 45 min in the dark. Gel pieces were washed twice with 50 mM TEAB, dehydrated again in ACN, and air-dried. Proteins were digested o/n (18 h, 37 °C) with sequencing-grade trypsin in 50 mM TEAB. Digestion was quenched with 1% trifluoroacetic acid (TFA), and peptides were sequentially extracted twice with 60% ACN/1% TFA, combining all supernatants. The pooled extracts were dried by vacuum centrifugation, reconstituted in 0.1% TFA, and desalted using C18 StageTips (Thermo Fisher Scientific) prior to LC–MS/MS analysis.

LC–MS analysis was performed using a Dionex UltiMate 3000 UHPLC system (Thermo Scientific) coupled to an Orbitrap Ascend mass spectrometer (Thermo Scientific). Peptides were loaded onto a C18 trapping column (Acclaim PepMap 100, 100 µm × 2 cm, 5 µm, 100 Å) at a flow rate of 10 µl/min and subsequently separated on an analytical C18 column (Acclaim PepMap C18, 75 µm × 50 cm, 2 µm, 100 Å) maintained at 50 °C using a 120-min linear gradient from 0–35% acetonitrile in 0.1% formic acid at 300 nl/min. The Orbitrap Ascend was operated in positive ion mode using a data-dependent acquisition (DDA) strategy with an expected chromatographic peak width of 30 s. Full MS scans were acquired in the Orbitrap at 120,000 resolution (m/z 375–1500) with quadrupole isolation, an AGC target of 4 × 10^5^, and automatic injection time. The most intense precursor ions (charge states 2–7, intensity > 1 × 10^4^) were selected for collision-induced dissociation (CID) at a normalised collision energy of 32%. MS^2^ spectra were acquired in the ion trap at turbo scan rate using a 0.7 m/z isolation window, AGC target of 1 × 10^4^, and automatic injection time. Dynamic exclusion was enabled for 45 s within ± 10 ppm of the precursor m/z, and single isotopes were excluded after one occurrence to improve proteome coverage.

Raw files were processed in Proteome Discoverer 3.0 (Thermo Scientific) using the Sequest HT search engine with Percolator validation. Spectra were searched against the CR ICC168 UniProt FASTA database, including a common contaminant database. Searches assumed full tryptic specificity with up to 2 missed cleavages. Carbamidomethylation of cysteine (+ 57.021 Da) was set as a fixed modification, while oxidation of methionine (+ 15.995 Da) and deamidation of asparagine or glutamine (+ 0.984 Da) were set as variable modifications. The precursor and fragment mass tolerances were 20 ppm and 0.5 Da, respectively. Peptide-spectrum matches were validated using a target-decoy strategy with Percolator, maintaining 1% FDR thresholds at the peptide and protein level.

### Explant cytokines

Distal colon tissue was excised, weighed, and incubated in RPMI 1640 medium with glutamine (Sigma; 100 μg/ml Sm, 100 U/ml penicillin) for 2 h at 37 °C. Tissue samples were then washed and incubated in complete RPMI (RPMI 1640 + glutamine, 10% FBS, 1 mM sodium pyruvate, 100μg/ml penicillin, 100μg/ml Sm; 1 ml/0.1 g of tissue) for 24 h at 37 °C and 5% CO_2_. Supernatants were centrifuged for 10 mins at 15,000 rpm to remove debris, and stored at − 70 °C until analysis. Cytokine concentrations were quantified using a custom LEGENDplex Mouse 13-plex cytokine panel (BioLegend), consisting of CCL3, CXCL1, G-CSF, GM-CSF, IFN-γ, IL-1β, IL-6, IL-10, IL-17A, IL-18, IL-22, IL-23 and TNF, according to the manufacturer’s instructions. Cytokine concentrations were acquired on a FACSCalibur flow cytometer (BD Biosciences), and data were analysed using LEGENDplex data analysis software (BioLegend).

### Colonic cell preparation for flow cytometry

Sections of distal colon 3.5 cm in length were excised and immediately transferred to cold Hanks’ Balanced Salt Solution (HBSS; Gibco) on ice. The tissue was opened longitudinally, cleaned of excess fat and washed twice in cold HBSS to remove luminal contents. To remove IECs, colons were incubated in 15 ml EDTA-DTT buffer (HBSS supplemented with 10 mM EDTA pH8.0, 1 mM DTT, 2% FBS) for 30 min at 37 °C shaking at 250 rpm. Samples were vigorously vortexed, blotted dry and rinsed in HBSS twice. Tissues were then digested in 5 ml digestion buffer (RPMI 1640 w/o L-glutamine supplemented with liberase (31.25 mg/ml), DNase I (50 μg/ml) and FBS (2%)) for 50 min in previous conditions. The resulting cell suspension was vortexed, filtered through 70 μm cell strainers and diluted with 20 ml cold HBSS. Cells were pelleted by centrifugation at 450 x *g* for 15 min at 4 °C and resuspended in 200 μl HBSS, transferred to a V-bottom 96 well plate and centrifuged for 4 min with previous conditions. Supernatants were discarded, and cells were washed in PBS w/o Ca and Mg (Gibco) before proceeding to staining.

### Colonic immune cell staining and flow cytometry

All staining steps were performed at 4 °C, protected from light. Cells were first stained in 100 μl of LIVE/DEAD™ Fixable Blue Dead Cell Stain (Thermo Fisher) diluted 1:500 in PBS for 10 min. Cells were washed once in FACS buffer (PBS supplemented with 2% FBS and 2 mM EDTA), centrifuged at 450 x *g* for 4 min, and the supernatant discarded. Cells were then incubated in 100μl Fc block solution consisting of FACS buffer supplemented with rat anti-mouse CD16/CD32 (clone ID 2.4G2, 1μg/ml; BDBiosciences) for 20 min. After washing, cells were stained with 30μl of antibody preparation (Supplementary Data 2; gating strategy for Fig. 2 from C57BL/6 mice at 8 dpi found in Supplementary Fig. 2C; gating strategy for B cell populations in C57BL/6 mice at 30 dpi found in Supplementary Fig. 4F) prepared in a 1:1 mix of FACS buffer and Brilliant Stain Buffer (BD Biosciences) and incubated for 30 min. For B cell analysis at 30 dpi, B cells were defined as a lineage-negative population lacking (CD4^−^CD8a^−^CD11b^−^TER119^−^FceRI^−^CD11c^−^GR1^−^NK1.1^−^F4/80^−^CD5^−^CD170^−^CD127^−^), representing cells devoid of other immune lineage markers included in the panel. Unstained cells and FMOs were used as controls. Cell analysis was carried out on a Cytek® Aurora Cytometer (Cytek Biosciences). Results were analysed using FlowJo v10 software (FlowJo LLC, BD).

### Gut-on-chip construction and infection

Master moulds for producing microfluidic chips were designed using Fusion 360 CAD software (AutoDesk, USA). One-layer microfluidic chips were produced first by mixing polydimethylsiloxane (PDMS; Sigma Aldrich, UK) and curing solution (Sigma Aldrich, UK) at a w/w ratio of 10:1. The PDMS/curing agent solution was degassed and poured onto master moulds, and then the elastomer mix was cured for 12 h at 65 °C. The cured polymer was then cut from the moulds, plasma treated for 1 min and bonded to glass coverslips. Caco-2 cells (HTB-37, ATCC, UK) were seeded on collagen-coated (50 μg/ml) chips at a density of 40 million cells/ml. After 24 h with no flow, chips were connected to syringe pumps and perfused with growth media (DMEM, 20% FBS) at a flow rate of 60 μl/hr.

After 4 days of perfusion, chips were removed from the flow systems and infected with either EPEC or ECV at an MOI of 10 for 4 h. Chips were then fixed with 4% paraformaldehyde for 15 min and labelled for intimin, zonula occludens-1 (ZO-1) and actin cytoskeleton. Images were taken with a confocal microscope with LSM capability and analysed using Icy Imaging software v3.

### Statistics

Experiments were conducted at least twice with 4-5 mice per treatment group, unless otherwise stated in the figure legends. Data from independent experiments were combined for statistical analyses. ELISAs calculations were performed on duplicate samples, and the mean value of the technical replicates was used for each sample. Statistical significance between two groups was determined by a two-tailed Student’s *t* test for normally distributed data. Comparisons among three or more groups were analysed by one-way ANOVA with indicated post-tests, or by Kruskal-Wallis test followed by Dunn’s test when data were not normally distributed. If required, values were log-transformed prior to one-way ANOVA, as indicated in the figure legends Statistics were calculated using GraphPad Prism 10.6.0 (GraphPad Software Inc.). Further statical details of experiments are described in the figure legends.

## Supplementary information

Supplementary Information

Description of Additional Supplementary File

Supplementary data 1

Supplementary data 2

Transparent Peer Review file

## Source data

Source data

## Data Availability

The mass spectrometry data generated in this study have been deposited in the ProteomeXchange partner repository database under accession PXD080490. The rest of the data that support the findings of this study are available within the main text, the Supplementary Information and the provided Source data. Source data are provided in this paper.
