# Supplementary material for: Design of a live-attenuated bacterial vaccine using effector network engineering
Source: Nat Commun. 2026 Aug 7;17:9629. doi: 10.1038/s41467-026-76138-7 (PMC13554080; doi:10.1038/s41467-026-76138-7)
Supplement: Supplementary file 1 [file 41467_2026_76138_MOESM1_ESM.pdf]

Supplementary Information

Design of a live-attenuated bacterial vaccine using effector network engineering

Jordan *et al*.

This file contains:

Supplementary Figure 1

Supplementary Figure 2

Supplementary Figure 3

Supplementary Figure 4

Legends for Supplementary Table 1 and 2

Supplementary Figure 1

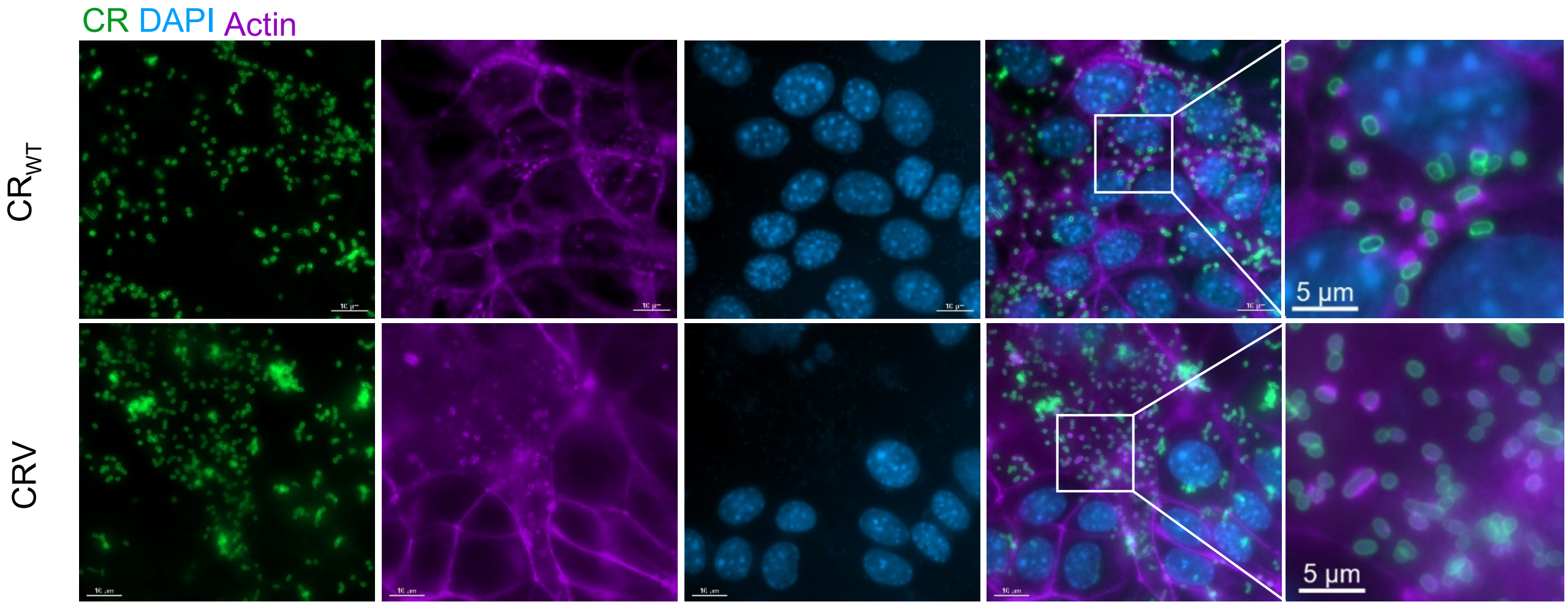

Supplementary Figure 1. CRV adheres to cells through A/E lesion formation in vitro. Representative images of WT CR and CRV in vitro infections of CMT-93 cells. Staining represents CR (green), actin (pink) and cell nuclei (DAPI). Scale bar = 10 mm and 5 mm (zoom). Representative images from 3 independent experiments.

Supplementary Figure 2

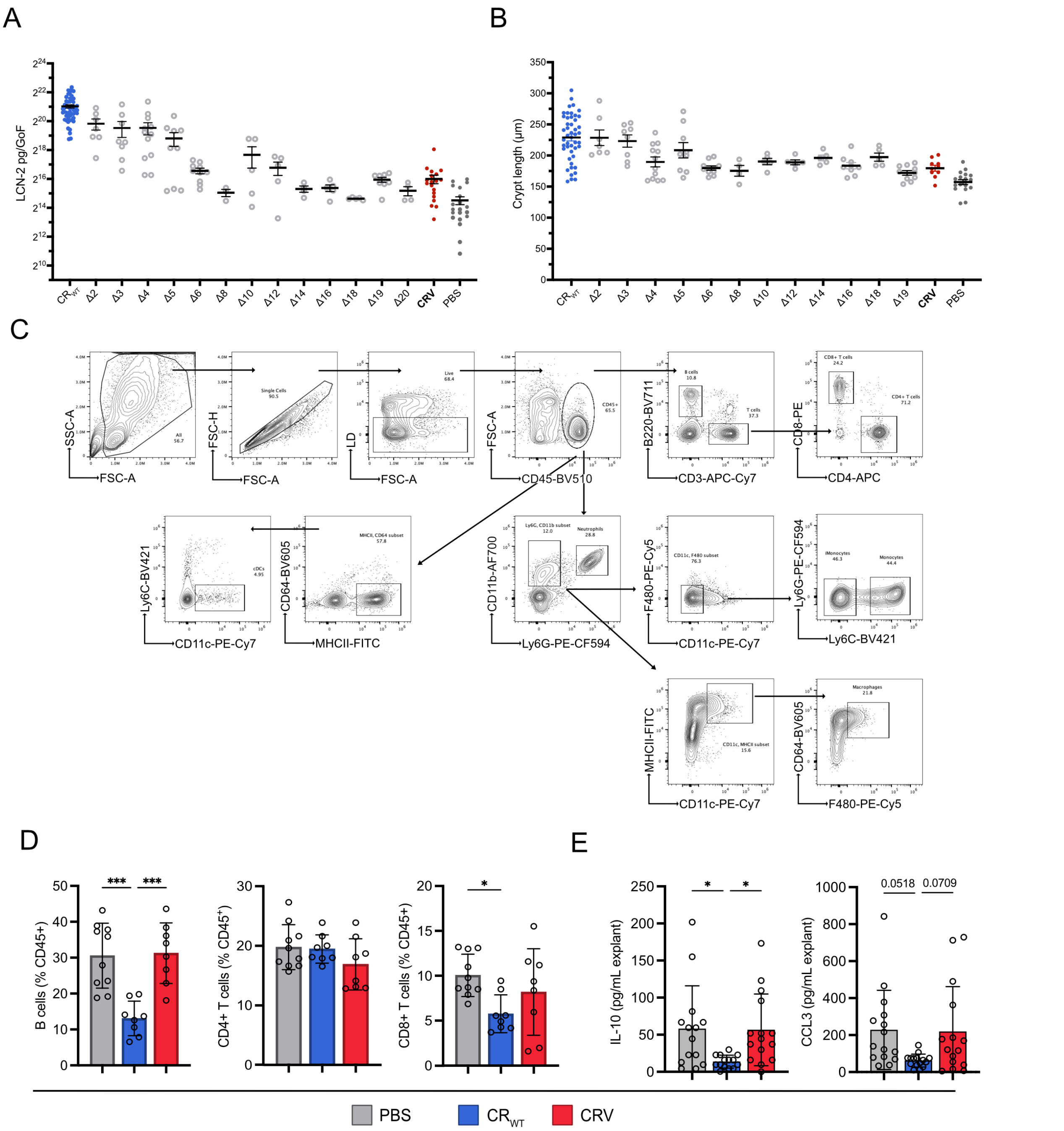

Supplementary Figure 2. Infection outcomes along the CRV deletion series and characterisation of CRV infection in C57BL/6 mice at 8 dpi. (A) LCN-2 levels and (B) colonic crypt length decrease along the CRV effector deletion series. Each dot represents value from individual mouse. Line represents mean ± SD. (C) Gating strategy used to differentiate $CD45^+$ cells within the colonic lamina propria. (D) Lymphocytes as a percent of $CD45^+$ cells and (E) explant cytokines within the colonic lamina propria. Each dot represents values for individual mice. Line represents mean ± SD. n = 2 (lymphocytes) or 3 (cytokines) biological replicates, 4-5 mice per group. Statistical significance was determined by one way ANOVA with Tukey's multiple comparison test including all pairwise comparisons. Only significant comparisons are shown, non-significant comparisons ($p>0.05$) are omitted; *$p<0.05$; ****$p<0.0001$. Source data are provided in the Source Data file.

Supplementary Figure 3

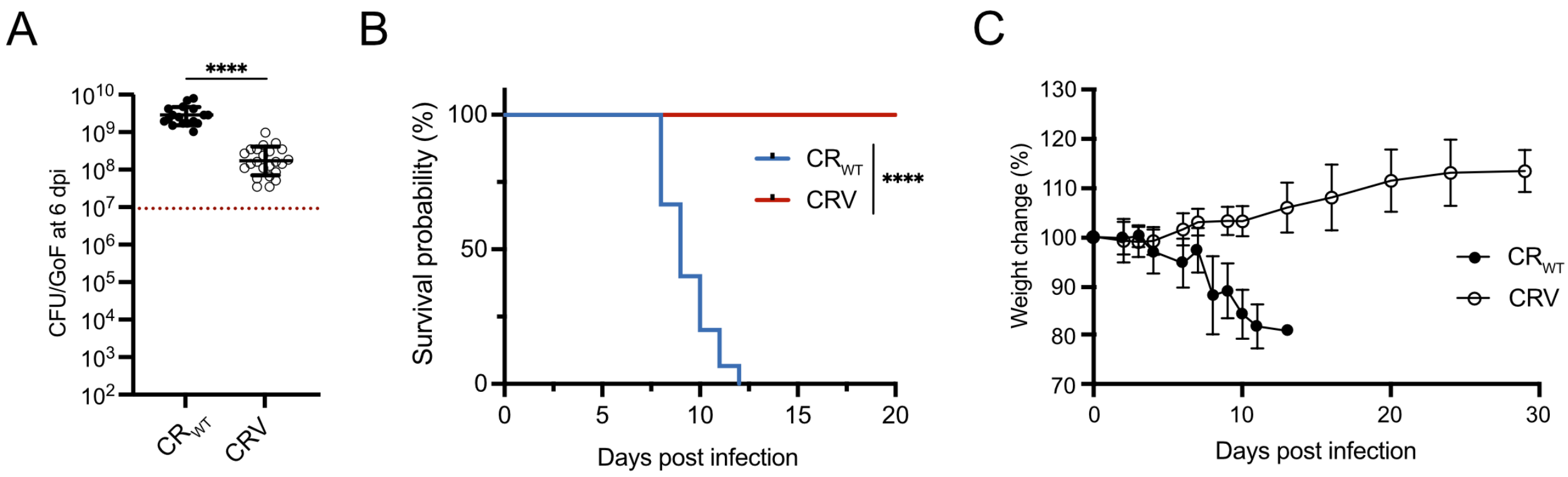

Supplementary Figure 3. C3H/HeN mice are not susceptible to CRV infection. (A) Enumeration of CFU/GoF at 6 dpi – infection peak in this mouse genetic background. (B) Survival curve of C3H/HeN mice infected with WT CR and CRV. (C) Mouse weight loss following WT CR or CRV infection. n = 3 biological replicates, 4-5 mice per group. dpi = days post infection. Statistical significance was determined by two-tailed Student's t test (A) or log-rank (Mantel-Cox) test (B). ****p<0.0001. Source data are found within the Source Data file.

Supplementary Figure 4

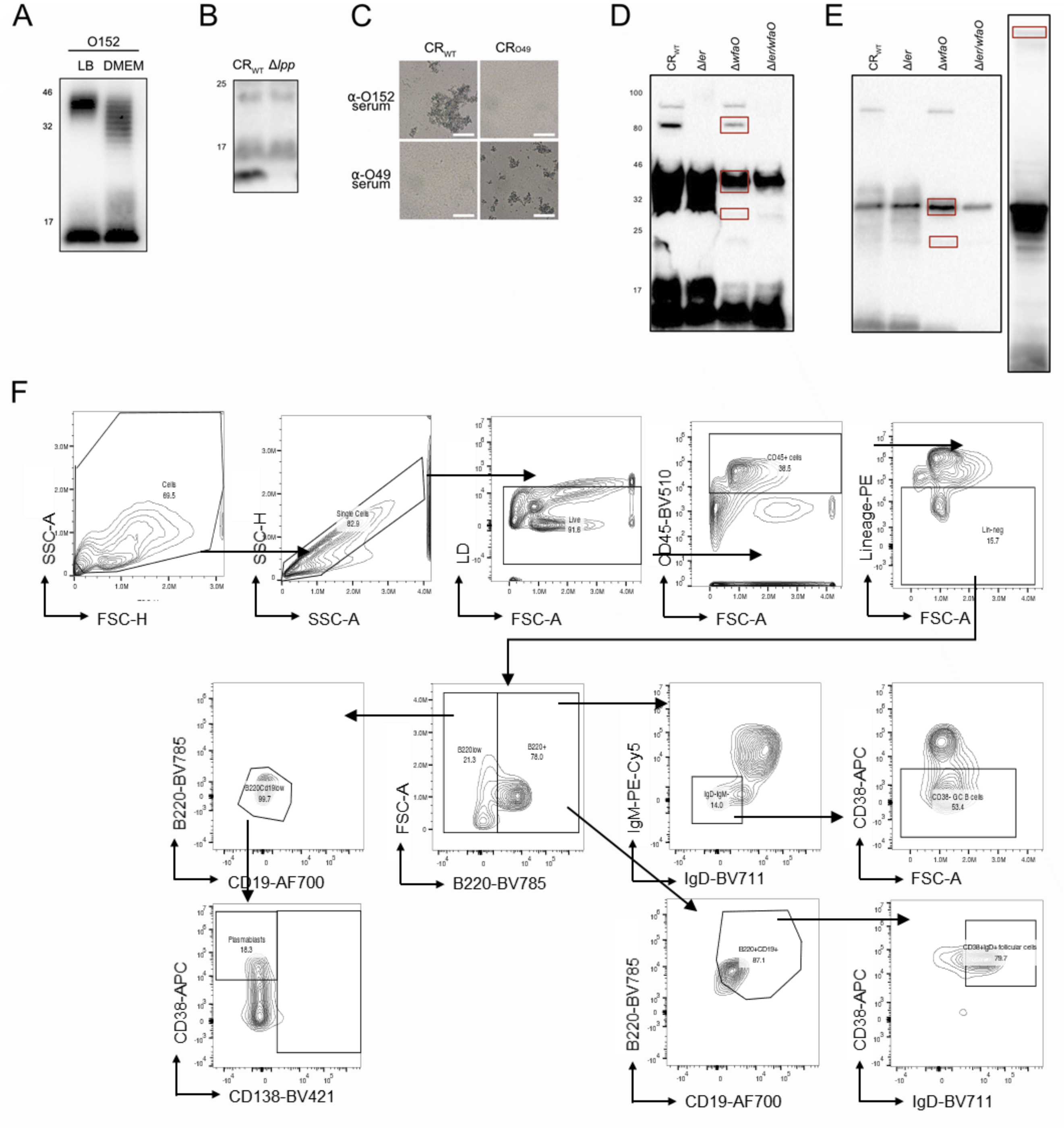

Supplementary Figure 4. Antigen discovery and characterisation. (A-B) Representative WB images confirming convalescent serum from WT CR-infected mice at 30 dpi is reactive to the O-antigen and Lpp. (C) Representative images of bacterial culture agglutination with anti-O152 and anti-O49 serum. Scale bar = 20 mm. (D) WB against various CR mutant bacterial lysates and (E) outer membrane preps identifying uncharacterised antigens, including outer membrane proteins ~30, 45 and 250 kDa in size and a Ler-regulated protein ~80 kDa. (F) B cell flow cytometry gating strategy used to identify B cell populations within the mouse lamina propria at 30 dpi. All blots are representative of at least two independent experiments. Agglutination images are representative of two assays.

Supplementary Table 1. Mass spectrometry identification of proteins from 30 kDa, 40 kDa, and 255 kDa bands. This table provides a comprehensive list of candidate proteins identified via bottom-up liquid chromatography-mass spectrometry (LC-MS/MS) from excised gel bands at approximately 30 kDa, 40 kDa, and 255 kDa. Individual tabs/sections break down the identified proteins by their respective molecular weight bands. Data fields include protein accessions, descriptions, sequence coverage, score thresholds, and the minimum number of unique peptides used for confident protein identification.

Supplementary Table 2. Inventory of experimental resources including bacterial strains, plasmids, primers, and antibodies. This table lists the critical biological and chemical reagents used across separate sheets. Key fields include species, strain/genetic background details, plasmid characteristics, exact oligonucleotide sequences for all primers, and comprehensive antibody details (target antigen, host species, clonality, clone/lot numbers, supplier, catalog number, and experimental dilutions).
