# Supplementary material for: Design of a live-attenuated bacterial vaccine using effector network engineering
Source: Nat Commun. 2026 Aug 7;17:9629. doi: 10.1038/s41467-026-76138-7 (PMC13554080; doi:10.1038/s41467-026-76138-7)
Supplement: Supplementary file 2 [file 41467_2026_76138_MOESM2_ESM.pdf]

Description of the Additional Supplementary Files

Supplementary Data 1: Mass spectrometry identification of proteins from 30 kDa, 40 kDa, and 255 kDa bands. This table provides a comprehensive list of candidate proteins identified via bottom-up liquid chromatography-mass spectrometry (LC-MS/MS) from excised gel bands at approximately 30 kDa, 40 kDa, and 255 kDa. Individual tabs/sections break down the identified proteins by their respective molecular weight bands. Data fields include protein accessions, descriptions, sequence coverage, score thresholds, and the minimum number of unique peptides used for confident protein identification.

Supplementary Data 2: Inventory of experimental resources including bacterial strains, plasmids, primers, and antibodies. This table lists the critical biological and chemical reagents used across separate sheets. Key fields include species, strain/genetic background details, plasmid characteristics, exact oligonucleotide sequences for all primers, and comprehensive antibody details (target antigen, host species, clonality, clone/lot numbers, supplier, catalog number, and experimental dilutions).
