# Supplementary material for: Design of a live-attenuated bacterial vaccine using effector network engineering
Source: Nat Commun. 2026 Aug 7;17:9629. doi: 10.1038/s41467-026-76138-7 (PMC13554080; doi:10.1038/s41467-026-76138-7)
Supplement: Supplementary file 3 [file 41467_2026_76138_MOESM5_ESM.pdf]

nature portfolio

Peer Review File

Design of a live-attenuated bacterial vaccine using effector network engineering

Corresponding Author: Dr Gad Frankel

This file contains all reviewer reports in order by version, followed by all author rebuttals in order by version.

Version 0:

Reviewer comments:

Reviewer #1

(Remarks to the Author)
I have no further comments and congratulate the authors on their excellent work.

Reviewer #2

(Remarks to the Author)
Overall, the authors have addressed the previous comments thoroughly, and the revision is greatly improved. The manuscript is clearer, the data are better contextualized, and most concerns have been satisfactorily resolved. I appreciate the additional experiments and clarifications provided.
Figure 6. However, the data presented in Figure 6 remain insufficient to support the claim that the ECV strain forms pedestals similarly to WT EPEC. The images appear to show predominantly bacterial adherence rather than well-defined pedestal structures. The fields are heavily overloaded with bacteria, most of which do not exhibit pedestals, making it difficult to clearly assess actin rearrangements. To strengthen this conclusion, the authors should repeat the experiment using a lower MOI and provide images in which actin is labeled in a color distinct from magenta, enabling clearer visualization of actin localization relative to the bacteria. Higher-resolution images are also needed, and a negative control strain incapable of pedestal formation (such as a Δtir mutant) should be included to validate the specificity of the observed structures.

Reviewer #3

(Remarks to the Author)
I congratulate the authors on a comprehensive revision of the manuscript and thank them for thoroughly addressing the questions posed. The work is of high quality. The conclusions are supported by the results presented. In this reviewer's opinion, the manuscript is suitable for publication in the high-impact journal, Nature Communications.

Open Access This Peer Review File is licensed under a Creative Commons Attribution 4.0 International License, which permits use, sharing, adaptation, distribution and reproduction in any medium or format, as long as you give appropriate credit to the original author(s) and the source, provide a link to the Creative Commons license, and indicate if changes were made.
In cases where reviewers are anonymous, credit should be given to 'Anonymous Referee' and the source.
The images or other third party material in this Peer Review File are included in the article's Creative Commons license, unless indicated otherwise in a credit line to the material. If material is not included in the article's Creative Commons license and your intended use is not permitted by statutory regulation or exceeds the permitted use, you will need to obtain permission directly from the copyright holder.
To view a copy of this license, visit https://creativecommons.org/licenses/by/4.0/

Response to reviewers' comments

Reviewer #1

Comment: I have no further comments and congratulate the authors on their excellent work.

Response: We thank the reviewer for their positive assessment of the work.

Reviewer #2

Comment 1: Overall, the authors have addressed the previous comments thoroughly, and the revision is greatly improved. The manuscript is clearer, the data are better contextualized, and most concerns have been satisfactorily resolved. I appreciate the additional experiments and clarifications provided.

Response: We thank the reviewer for their original comments which indeed improved the clarity and quality of the manuscript.

Comment 2: The data presented in Figure 6 remain insufficient to support the claim that the ECV strain forms pedestals similarly to WT EPEC. The images appear to show predominantly bacterial adherence rather than well-defined pedestal structures. The fields are heavily overloaded with bacteria, most of which do not exhibit pedestals, making it difficult to clearly assess actin rearrangements. To strengthen this conclusion, the authors should repeat the experiment using a lower MOI and provide images in which actin is labeled in a color distinct from magenta, enabling clearer visualization of actin localization relative to the bacteria. Higher-resolution images are also needed, and a negative control strain incapable of pedestal formation (such as a Δtir mutant) should be included to validate the specificity of the observed structures.

Response: We have now split Fig. 6 and the gut on the chip is shown in a new Fig. 7. We have improved the resolution and added a zoom inset. The Figure clearly shows that ECV forms pedestal and already contain the necessary WT EPEC control. By definition, a *tir* mutant doesn't trigger pedestal formation and there is no need to show it again, the experiment aimed to compare WT and ECV.

Reviewer #3 (Remarks to the Author):

Comment: I congratulate the authors on a comprehensive revision of the manuscript and thank them for thoroughly addressing the questions posed. The work is of high quality. The conclusions are supported by the results presented. In this reviewer's opinion, the manuscript is suitable for publication in the high-impact journal, Nature Communications.

Response: We thank the reviewer for their strong support of the study.
